# Systematic Study of Different Types of Interactions in α-, β- and γ-Cyclodextrin: Quantum Chemical Investigation

**DOI:** 10.3390/molecules29102205

**Published:** 2024-05-08

**Authors:** Imre Bakó, László Jicsinszky, Szilvia Pothoczki

**Affiliations:** 1 HUN-REN Research Centre for Natural Sciences, Magyar Tudósok Körútja 2, H-1117 Budapest, Hungary; 2 Dipartimento di Scienza e Tecnologia del Farmaco, University of Turin, Via P. Giuria, 9, 10125 Turin, Italy; laszlo.jicsinszky@unito.it; 3 HUN-REN Wigner Research Centre for Physics, Konkoly Thege M. út 29-33, H-1121 Budapest, Hungary

**Keywords:** cyclodextrins, H-bond, vibrational frequencies, Atom in molecules, Natural Bond Orbital, Chemical Energy Component Analysis

## Abstract

In this work, comprehensive ab initio quantum chemical calculations using the DFT level of theory were performed to characterize the stabilization interactions (H-bonding and hyperconjugation effects) of two stable symmetrical conformations of α-, β-, and γ-cyclodextrins (CDs). For this purpose, we analyzed the electron density using “Atom in molecules” (AIM), “Natural Bond Orbital” (NBO), and energy decomposition method (CECA) in 3D and in Hilbert space. We also calculated the H-bond lengths and OH vibrational frequencies. In every investigated CD, the quantum chemical descriptors characterizing the strength of the interactions between the H-bonds of the primary OH (or hydroxymethyl) and secondary OH groups are examined by comparing the same quantity calculated for ethylene glycol, α-d-glucose (α-d-Glcp) and a water cluster as reference systems. By using these external standards, we can characterize more quantitatively the properties of these bonds (e.g., strength). We have demonstrated that bond critical points (BCP) of intra-unit H-bonds are absent in cyclodextrins, similar to α-d-Glcp and ethylene glycol. In contrast, the CECA analysis showed the existence of an exchange (bond-like) interaction between the interacting O…H atoms. Consequently, the exchange interaction refers to a chemical bond, namely the H-bond between two atoms, unlike BCP, which is not suitable for its detection.

## 1. Introduction

**Figure 1:**
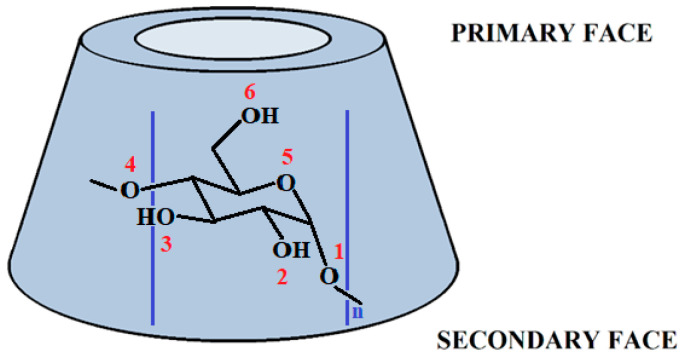
Schematic representation of cyclodextrins and atomic numbering scheme of α-D-glucopyranose unit. The number of O atoms is marked with red color. One α-D-glucopyranose (α-D-Glcp) unit can be found between the blue lines.

Cyclodextrins (CDs) are macrocyclic oligosaccharides built up from α-d-glucopyranose (α-d-Glcp) units, connected by α(1→4) glycosidic linkages [1,2,3,4,5]. A schematic representation of these molecules is shown in Figure 1, together with the α-d-glucopyranose molecule that builds up these molecules, the O’s are numbered according to their identity. These systems can be produced by the fermentation of starch. Their most abundant forms are α-, β-, and γ-CDs, consisting of 6, 7, and 8 glucose units. The shape of the CD can be characterized as a truncated cone with the primary OH (or hydroxymethyl) groups at the narrower rim of the cavity and the secondary hydroxyl groups at the wider side. The cavity size of CD is in the range of 5.7–9.5 Å^3^. Due to their structural properties, CD molecules can easily encompass different molecules in or near their cavity, commonly referred to as inclusion complexation. This well-known feature allows for the use of CDs in many areas, like the pharmaceutical and food industry, analytical chemistry, biotechnology, and agriculture [6,7,8,9,10].

Hyperconjugation, H-bonds and electrostatic interactions are decisive factors regarding the structural rigidity of the CD molecules, which plays an important role in inclusion complex formation [3,11,12,13,14]. This study highlights the characteristics of the H-bonded connections within the molecule due to their key roles in rigidity [3,11,12,13,14]. These intramolecular H-bonds can be classified into two types: in the “inter-unit” case, H-bonds are between two α-d-glucose units, and in the “intra-unit” case, H-bonds are formed within α-d-glucose units.

Gas phase calculations showed that a strong inter-unit H-bond exists between the primary hydroxyl groups (marked as 6 in Figure 1) in the most stable conformer of the CDs [12,13,14]. The primary OH groups can also form hydrogen bonds (H-bonds) to the pyranose ring oxygen of the adjacent glucose unit [12,13,14]. On the other hand, the H-bond between the secondary hydroxyl groups (marked as 2 in Figure 1) at the wider rim is weaker than those between the primary hydroxyl groups at the narrow rim [13]. In crystalline form, the secondary OH group of two neighboring units forms an inter-unit H-bond with decreasing H-bond length as the number of sugar units increases [3]. However, less attention was paid to elucidating the role of the intra-unit H-bond interactions in structural rigidity, especially O2H2_n_…O3_n_, which has a decisive role in forming the H-bond linkages extending along the wider rim.

On this basis and considering earlier results [3,11,12,13,14], in our present work the four most significant types of H-bond were monitored according to which parts of the CD molecules participate in the formation of the H-bond: (1) primary inter-unit H-bond (O6H6_n−1_…O6_n_): the H-bond forming between the primary OH groups; (2) secondary intra-unit H-bond (O2H2_n_…O3_n_): the H-bond forming between the secondary OH groups in the same glucose subunit; (3) secondary inter-unit H-bond (O3H3_n_…O2_n−1_): the H-bond forming between the secondary OH groups of the neighboring glucose subunits; (4) primary inter-unit H-bond (O6H6_n−1_…O5_n_): the H-bond forming between the primary OH, group and O5 (pyranose) oxygen. It is worth noting, that the orientation of the inter-unit hydrogen bonds both in the wider rim and the narrower rim can be clockwise or counterclockwise [13,14], but we do not take into account this differentiation.

**Figure 2:**
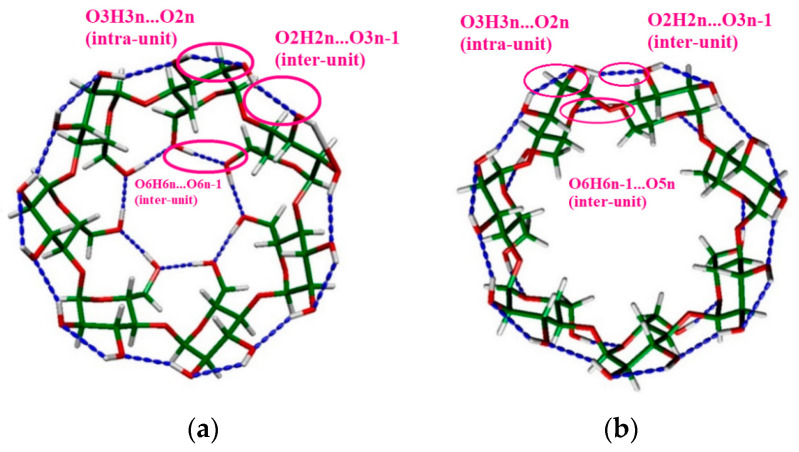
(**a**) “Closed” and (**b**) “Open” structures in β-CD.

We focus on two regular structures, hereinafter referred to as the “Closed” structure (cf. Figure 2a for β-CD), in which both the primary OH groups (O6H6_n_…O6_n−1_) along the narrower rim and the secondary OH groups (O2H2_n_…O3_n−1_ and O3H3_n_…O2_n_) along the wider rim form a cyclic, closed, H-bonded ring. For all three CDs, this conformer was proven to be the most stable one. In the other case, hereinafter called “Open” (Figure 2b), only the secondary OH groups (O2H2_n_…O3_n−1_, O3H3_n_…O2_n_) form a closed cyclic H-bonded ring along the wider rim. Furthermore, the primary OH groups are linked to the ring oxygen in the neighboring glucose unit (O6H6_n−1_…O5_n_). The presentations of the two studied structures (“Open” and “Closed”) indicating the above-mentioned bonds for β-CD are shown in Figure 2.

Vibrational spectroscopic methods, especially IR spectroscopy are widely used tools to investigate H-bonding [15,16,17,18]. The shift of the peak positions and the intensity changes indicate the existence and strength of the H-bond connections [15,16,17,18]. An interacting OH bond is shifted (usually towards lower frequencies, that is, red-shifted) compared to the frequency of free non-interacting OH bonds. Furthermore, a more significant shift in the frequencies indicates a stronger bond [15,16,17,18].

Some previous studies on IR bands of β-CD [19,20] systems revealed the separability of IR absorptions of the three different types of OHs in CDs. In a later publication, Egyed and Weiszfeiler also pointed out a red shift of the H-bonded hydroxymethyl group in the 3500 cm^−1^ region [20], where the salt formation between the secondary hydroxyl groups and copper (II) allows a better separation of the signals of the primary and secondary OHs. In crystalline CDs, water molecules are usually also present in the CD cavity, and it is experimentally impossible to separate the H-bond absorption bands between the included water and the CD hydroxyls. Consequently, the experimental spectra show the combination of signals from all possible H-bond variations [3].

Our work focuses on the two most symmetrical conformers of the three most abundant (α-, β-, and γ-) CDs in the gas phase. First, we calculate the geometrical and vibrational quantities, namely the H-bond lengths (Section 2.2) and vibrational frequencies (Section 2.3), to establish links to the experimental results and, in this way, to gain information about the presence and the strength of the above-listed intra- and inter-unit H-bonds. On the other hand, for the same purpose, we use various approaches, namely the “Atoms in molecules” (Section 2.4), the “Natural Bond Orbital” (Section 2.5) and the ‘‘Chemical Energy Component Analysis’’ (Section 2.6) derived from quantum chemical methods for our investigation. For all these approaches, we endeavor to discuss their advantages and limitations in detail.

The most accepted definitions of H-bonding use the “Atoms in molecules” (AIM) approach, which uses the topological properties of the electron density to estimate the existence and strength of H-bonds [21,22,23,24,25]. Some studies seriously questioned this approach [26,27,28,29,30,31,32,33,34,35,36,37] because in many cases, for example, for monosaccharides [27,28,29,30,31,32,33,34] and even for ethylene glycol [35,36,37], the AIM method cannot detect any bond as a critical bond between the secondary OH groups, despite the spectroscopic measurement assuming the existence of H-bonds between these groups [38]. On the other hand, Lane et al. [39] revealed that the absence of a Bond Critical Point (BCP) in the ethylene glycol g+Gg- conformation does not provide a direct consequence for the missing H-bond. They demonstrated that examining the BCP alone for an existing H-bond is insufficient, but it is also necessary to explore the environment of the assumed BCP (whether it exists or not). In these special cases, the X…H distance is significantly longer (around 2.5 Å), and H-bonding is less linear than in the case of water clusters.

The “Natural Bond Orbital” (NBO) analysis is also usually used to characterize the H-bonds [40,41]. The size of the delocalization interaction from the lone pair orbital to the X-H nonbonding orbital can be applied to quantify the strength of the H-bond and showed a good correlation with data from AIM analyses [42,43]. In the case of monosaccharides, it is shown that the NBO calculation gives a significant delocalization energy contribution from the lone pair of the pyranose oxygen (LPO) to the antibonding orbital of the CO1 bond (BD*CO) [44,45,46,47,48,49]. This fact was used to support the explanation of the anomeric effect, which is related to the conformational stability of the monosaccharide.

It is well-accepted that several different types of energy components are involved in the formation of hydrogen bonds. The ‘‘Chemical Energy Component Analysis’’ (CECA) is a suitable tool to decompose the total energy into atomic and diatomic energy contributions. The second (diatomic) term can be decomposed into electrostatics, exchange effects, diatomic overlap, and atomic basis extension terms [50,51,52,53,54,55]. Among these, electrostatic and orbital interactions are perhaps the most important. Energy contributions from orbital interactions are the formation of chemical bonds, which originate primarily from exchange interactions. It has been shown previously [56], that despite the energy values obtained using CECA analysis being too high, significantly larger than the energy of a real chemical bond, their ratio provides information on the importance of each interaction contribution. It is important to emphasize that this bonding character of H-bonds is not explicitly described in the AIM description, which investigates the topological properties of the electron density. To reveal the existence of weak chemical bonds (such as those coming from exchange interactions) the Mayer bond order and the CECA analyses are used. Here, we would like to note that a similar type of analysis based on a 3D decomposition of electron density has already been developed (Interacting Quantum Atoms, IQC) [56,57,58,59,60,61,62,63] and has recently become very popular, but similarities and dissimilarities to the CECA method have also been discussed in detail [62,63]. Comparison between CECA and the Ziegler–Rauk energy decomposition scheme (EDA), shows that both methods give chemically meaningful results despite their differences [55].

Finally, we must highlight that ethylene-glycol, α-d-Glcp and water clusters were used as reference systems to pinpoint the origin of the above-mentioned interactions and gauge their importance. Ethylene glycol, is one of the simplest systems, which contains the O-C-C-O motif (c.f. O3-C3-C2-O2 in Figure 1), while α-d-Glcp is the basic unit of CDs.

## 2. Results and Discussion

### 2.1. Benchmark Calculation for Energy Difference between the Two Conformers of CD

The total energy difference between the “Open” and the “Closed” form of the investigated CD systems was calculated by three different DFT approaches. The results are shown in Table 1.

It can be established for all three methods (BLYP/D3, ωB97XD, and M06-2X/D3) that the difference in the calculated (total) energy is the largest for α-CD and the smallest for γ-CD. This tendency does not change, even when the calculation on the same geometry was performed at a significantly higher level of theory (LNO-CCSD(T)/cc-pVTZ). The results based on the other approaches (BLYP/D3, ωB97XD, and M06-2X/D3) differ only slightly (ca. 3–4 kcal/mol) from this most accurate calculation.

It is worth noting here that by performing the same calculations for various conformers of α-d-Glcp, the difference (between LNO-CCSD(T) versus other different types of DFT approaches) was derived to be ca. 1 kcal/mol [30,31].

### 2.2. O-H Bond Lengths

When studying the strength of the H-bond, we calculated the OH…O intramolecular (intra- and inter-unit) H-bond distances for the three CDs for both symmetrical structures applying three different DFT methods (BLYP/D3, ωB97XD, and M06-2X/D3) that optimize the DFT/6-311** level. The results presented in Table 2 show that the calculated values are almost independent of the DFT method used. Therefore, in the following sections, only results arising from BLYP/D3 are presented.

The shortest H-bonding distances in all three CDs were between the primary OH groups (O6H6_n−1_…O6_n_). It is worth reiterating here, that this bond type is only in “Closed” type systems. Bond lengths of this type increase with the number of glucose units. The difference in the H-bond lengths is larger between β- and γ-CDs than between α- and β-CDs. In “Open” systems, the primary OH does not form an H-bond with another primary OH group, but does with the ring O of the neighboring unit (O6H6_n−1_…O5_n_). These H-bond lengths are significantly longer than those between the primary OHs (O6H6_n−1_…O6_n_) in “Closed” systems.

The intra-unit H-bond lengths of the secondary OH groups (O3H3_n_…O2_n_) in the “Closed” systems, which are almost the same magnitude for all the studied CDs, are longer than the inter-unit H-bond lengths (O2H2_n_…O3_n−1_).

The longest secondary inter-unit H-bond lengths were found in the α-CD. There are no significant differences in this type of distance between the β-CD and γ-CD. These results are consistent with previous results [12].

It is worth comparing the intra-unit H-bond lengths (O2H2_n_…O3_n_) of CDs (2.465 Å for α-CD and β-CD, and 2.468 Å for γ-CD) with those in our reference systems (ethylene glycol and α-d-Glcp). These distances were found to be shorter in both reference systems, namely 2.28 Å in g+Gg- ethylene glycol and 2.41 Å in the most stable α-d-Glcp conformer, than in any of our studied cyclodextrins.

### 2.3. OH Vibration Frequencies

The OH vibrational frequency is suitable for characterizing interactions between different OH groups. The calculated vibration frequencies are presented in Table 3.

For the most stable conformers (“Closed”), the vibration frequencies of the primary OH bonds are found at a significantly lower frequency (by more than 100 cm^−1^) than in the less stable conformer (“Open”). We need to remark, that the primary inter-unit H-bonds in the “Closed” type conformer are between two primary OH groups, while in the “Open” type conformer they are between the primary OH group and the pyranose oxygen. Moreover, where the primary OH is not bonded, the calculated vibrational frequency is significantly higher than that of the bonded (ca. 3684–3695 cm^−1^). A similar trend was observed with respect to the secondary inter-unit interaction, the vibrational frequency in the “Closed” system appears at lower frequencies than that in the “Open” one. The frequency difference is approximately 30–40 cm^−1^.

In the case of inter-unit interaction related to the secondary OH groups, the vibrational frequency appears at lower frequencies than in intra-unit ones, suggesting the inter-unit interactions are stronger. This statement applies to both conformers. These differences are ca. 120–140 cm^−1^ for the “Closed” conformer and ca. 70–90 cm^−1^ for the “Open” one.

**Figure 3:**
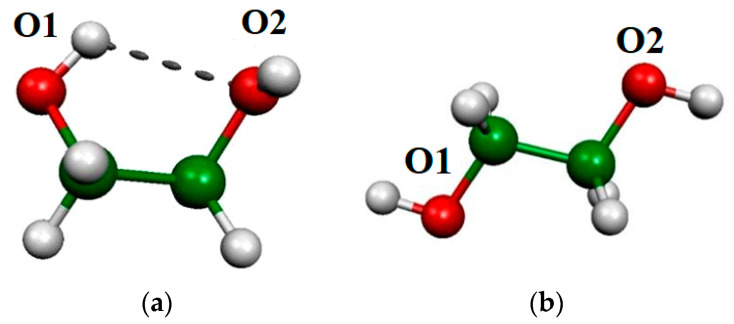
(**a**) The g+Gg- and (**b**) the tTt conformers of ethylene glycol.

Ethylene glycol and α-d-Glcp are good candidates for a deeper understanding of the intra-unit OH vibrations. In the case of ethylene glycol, two conformers (Figure 3) were studied. In the absence of OH…O H-bonds, the vibrations belong to the O1H and O2H groups of tTt and the O2H of g+Gg- of the ethylene glycol can be considered free OH vibrations, however the O1H vibrations in the g+Gg- conformer are not classified as free. Our results show (Table 4), that the vibrational frequency of the bonded O1H of the g+Gg- conformer is shifted by 40–80 cm^−1^ toward the lower frequencies compared to free OH vibrations.

**Figure 4:**
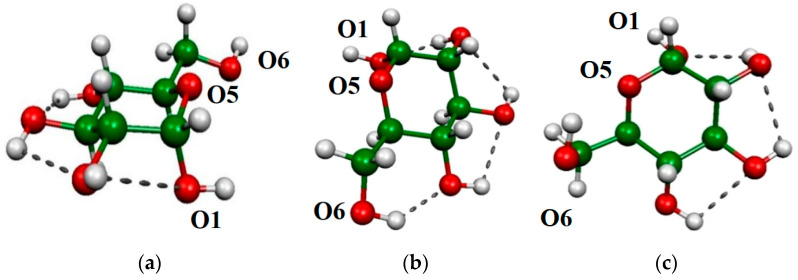
(**a**) The G-t/cc/t and (**b**) the Tg+/cc/t and (**c**) the G-g+/cc/t conformers of the of α-d-Glcp.

In the ‘G-t/cc/t’ (Figure 4a) conformer of α-d-Glcp O1H and O6H, while in the other two conformers (Figure 4b,c), only O1H can be considered as free groups without any significant interaction with the other part of the molecule. The vibrational frequency of the free vibrations was in the range of 3682–3693 cm^−1^ (Table 5). Compared to this, the vibrational frequency of the bonded OH is shifted by 40–90 cm^−1^ to the lower frequencies. These frequency shifts were explained [34] by the existence of an H-bond in glucose.

Based on the results of reference systems, our calculations indicate that the frequency shift caused by the intra-unit interaction (in the direction of lower frequencies) is in the range that can be considered an H-bonded state in these systems.

It is worth pointing out here, that the calculated OH vibration frequencies for the (H_2_O)_50_ water cluster, using a similar method, fall within the range of 3000–3500 cm^−1^. These vibrational modes are strongly coupled and cannot be described by such vibrations that can be localized to a water molecule. For a water monomer, these modes are 3659 cm^−1^ and 3756 cm^−1^. Note that, in the case of intra- and inter-unit OH vibrations of CDs, the redshift of the OH vibrations are significantly smaller than that of the water clusters.

### 2.4. Atoms in Molecules (AIM) Analyses

One of the most commonly used methods for discovering the existence of H-bonding is the AIM method described by Popelier [24]. This method derives from the features related to the topological properties of the electron density. In general, the electron density at the bond critical point (BCP) is also used to characterize the strength of the interactions [22,23,24]. In the case of H-bonding, this electron density is usually between 0.005–0.05 [e/Å^3^] [22,25]. It has already been shown [26,27] that this method is unsuitable for detecting the intramolecular H-bonds of the g+Gg- ethylene-glycol and the α-d-Glcp [35,36,37,38,39]. When examining intra-unit H-bonds, no critical point was found in CDs either, as expected, due to the weaknesses in the AIM method [35,39].

We calculated the average electron density (RHO(BCP)) at the BCP for the inter-unit OH bonds in the following cases, where we found the bond critical point: O6H6_n−1_…O6_n_ (primary inter-unit H-bond between two primary OH groups), O6H6_n−1_…O5_n_ (primary inter-unit H-bond between the primary OH group and O5 ring oxygen) and O2H2_n_…O3_n−1_ (secondary inter-unit H-bond between two secondary OH groups). The results are presented in Table 6 for the two conformers of the three studied CDs.

In the case of the three “Closed” CD conformers, the RHO(BCP) connecting to the secondary inter-unit H-bond differ only slightly from each other, but these values are significantly smaller than the corresponding ones in the “Open” systems. For the same interactions among the “Open” conformers, the smallest RHO(BCP) belongs to the β-CD. The RHO(BCP) associated with the primary inter-unit H-bonds, which exist only in the “Closed” conformers, is the largest in α-CD and decreases as the number of glucose units increases. These conclusions are in line with earlier results [35,39].

**Figure 5:**
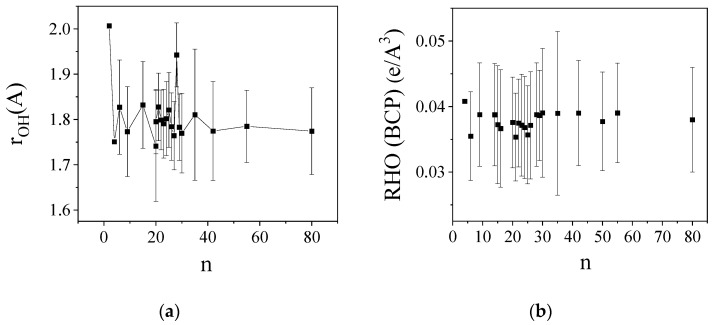
(**a**) The average bond length in water clusters as the function of cluster size (n) (Å), (**b**) The average electron density at BCP (e/Å^3^).

The average bond lengths and the average electron densities at the BCP were calculated in water clusters of various sizes ((H_2_O)_2_–(H_2_O)_80_). The results are presented in Figure 5. These values stabilize around a value of *n* between 8–10, though with a high standard deviation. The reason for the large standard deviation is likely due to the fact the electronic properties of water molecules (such as charge and dipole moment) are significantly influenced by their H-bonded environment. This means that the number of water molecules that form hydrogen bonds on either the donor or acceptor side can have a significant impact [64,65]. It appears that, at least for water clusters, an almost linear correlation exists between bond distances and densities at BCP (Figure S2). It can be stated that the H-bonds formed between the primary OH groups of the “Closed” structure, which proved to be the strongest H-bond, have an approximately identical electron density (RHO(BCP)) as those occurring in the water clusters. On the other hand, the RHO(BCP) for a secondary inter-unit H-bond is about 20–25% smaller. This fact reveals that this type of H-bond is weaker than the H-bond in the water cluster.

### 2.5. Natural Bond Orbital (NBO) Analyses

One of the characteristic features of H-bonding is the size of delocalization from an oxygen lone-pair orbital (LPO) to the nonbonding OH (BD*OH) orbital. The delocalization energy from second-order perturbation theory is suitable to describe the magnitude of this interaction. In general, if the delocalization energy is high, then the H-bond is also stronger (at least for the configurations in the energy minimum) [42,43]. The calculated delocalization energies of inter-unit H-bonds can be found in Table 7 for all studied CDs. We performed the calculations for both lone pairs of oxygen (LP(O1) and LP(O2)). Note here that we do not always get a contribution from both pairs of electrons (β-CD in “Closed” structure). In the case of the intra-unit H-bonded interaction, these delocalization terms are smaller than 0.05 kcal/mol.

In each CD, the delocalization energy between the primary OHs (O6H6_n−1_…O6_n_) in the “Closed” conformer is significantly higher than in the other cases. Among them, the weakest interactions can be seen in the case of γ-CD, while the strongest are for β-CD. The second strongest interactions are in the “Open” conformers between primary OH and pyranose O (O6H6_n−1_…O5_n_), the associated delocalization energy is significantly higher for α-CD than for the other two CDs (β-CD and γ-CD). Concerning the secondary inter-unit H-bonds, the delocalization energy is almost the same for the two conformers of α-CD. In the case of β-CD and γ-CD, the energy of the “Open” structure is larger than the “Closed” one.

It is important to emphasize that in all studied CDs, the energy of the interactions between the same α-d-Glcp unit’s O2H2 and O3, known as an intra-unit H-bond, was less than 0.1 kcal/mol. Similar NBO analysis for the intra-OH interaction in ethylene glycol resulted in approximately 1.2 kcal/mol. In the analysis of α-d-Glcp, no delocalization term was detected, which would have suggested the existence of H-bonding.

**Figure 6:**
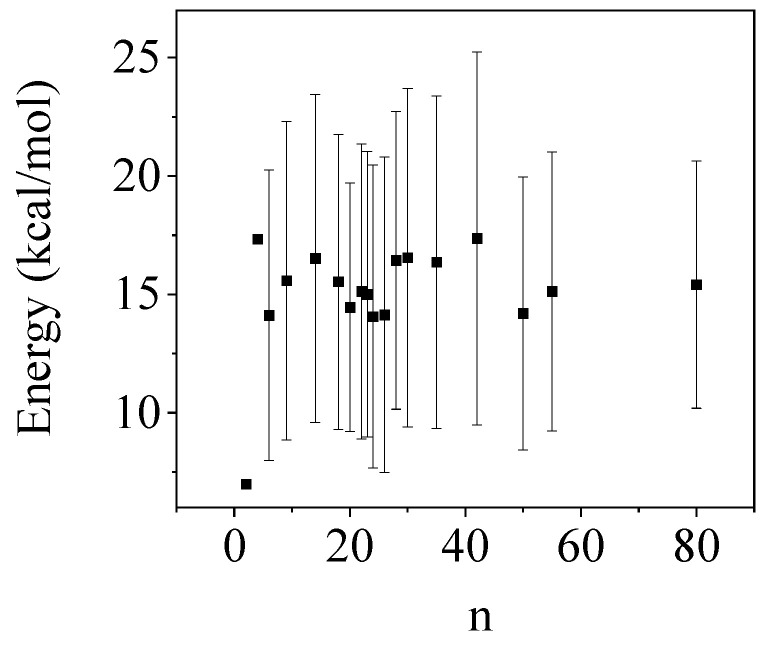
Average delocalization energy (kcal/mol) for water clusters as a function of cluster sizes (n).

The water dimer had an energy value of 7.0 kcal/mol, as presented in Figure 6. For water clusters with more than two molecules, the energy term increases significantly, all values exceed 14 kcal/mol. These findings show that the strength of H-bonding between different glucose units is considerably weaker than that of water clusters. These results agree well with those obtained for AIM calculations.

The stereoelectronic effect is well known to explain certain conformational properties of heterocyclic systems. This effect has long been applied in carbohydrate chemistry; the electronegative substituent attached to the C1 carbon atom determines a more stable conformation in the axial than in the equatorial position [54,55]. One of the most accepted explanations is the hyperconjugation interaction between the pyranose O and the C1X non-bonding orbital [56,57]. Several other explanations link this phenomenon mainly to electrostatic interactions (dipole–dipole, electron–electron repulsion) or unconventional H-bond formation. Delocalization energy is a frequently used quantity to examine the strength of hyperconjugation. In the CDs, there are two types of endocyclic oxygens: in positions O1 (glycosidic) and O5 (pyranose). The most significant delocalization energy contributions for all studied CDs (for both investigated conformers) and also the most stable conformer of α-d-Glcp are shown in Table 8).

For all studied CDs, the delocalization energy is largest in the position of the glycosidic oxygen in the case of the LPOBD*CO interactions. In the case of the “Closed” conformers, this energy decreases smoothly as the number of glucose units increases.

The delocalization energy assigned to the BD*CO orbital associated with the electron pair of O5, is significantly lower than that of O1. The delocalization energy related to this process increases with size in both conformations. These delocalization energies (O1 and O5) in the “Closed” conformer are comparable to the delocalization energy characteristic of the H-bond between the primary OH groups (c.f. LP(O1)O6H6…O6 values in Table 7).

The other significant delocalization terms assigned to the pyranose oxygen are smaller than the values discussed above. The delocalization energy characteristic of inter-unit H-bonds is significantly lower (c.f. LP(O1) O3H3…O2 values in Table 7) than these members.

The delocalization energy belonging to the LPO5BD*C1O1 orbit in α-d-Glcp is somehow higher than the value obtained in any CDs.

### 2.6. Bond Order and CECA

The interaction between the atoms that make up the molecule can also be characterized using the Hilbert space. In this case, we can use basis functions localized on the atoms for this purpose. Among these methods, the most frequently used is Mayer’s bond order analysis [50,51]. This quantity was shown to be closely related to the exchange interaction, which is connected to the covalent character between two atoms. However, it has already been shown [50] that it is not suitable (extremely high) for estimating the binding energy between atoms, as they give a very significant contribution even in the case of short distances. In these cases, an energy resolution method developed by Hamza and Mayer [52] and improved by other authors [53,54,55,56,57], results in a significantly better approximation. The interaction energy between the two atoms can be decomposed into electrostatic, exchange and overlap interactions. The exchange interaction always characterizes the formation of an attractive chemical bond. The overlap interaction can be both attractive and repulsive (Pauli repulsion) and includes all non-exchange interactions between electron clouds. The characteristic quantities for these specific interactions (probable H-bond) for α-, β- and γ-CD and the two reference systems (ethylene-glycol and α-d-Glcp) are shown in Table 9. We found a measurable exchange contribution, which unambiguously proves the existence of a quantum chemical bond between two secondary OH groups within the same glucose unit.

The results of Table 9 for the most stable CD conformers (“Closed”) are in line with our earlier statement based on our calculations of the length of the primary inter-unit H-bond (c.f. Section 2.2) that the H-bond length increases, that is, the strength of the H-bond decreases as the number of sugar units increases: both the bond order decreases, and the absolute value of all of the energy contributions (electrostatic and quantum chemical) decreases with the increasing number of sugar units. The two quantum chemical (exchange and overlap) contributions are almost identical to each other. According to this, we underline that the role of the quantum chemical contribution is the largest in the case of α-CD.

At the same time, in the case of secondary inter-unit H-bonds, the largest value was observed in the case of β-CD for all three types of interaction contributions. The ratio between inter- and intra-unit secondary H-bonds for the exchange and the overlap terms is around two. These exchange terms are significantly lower than the corresponding values of O6H6_n_…O6_n−1_ interactions. On the other hand, for all calculated quantities of “Open” systems, local extrema (minimum or maximum) were found in the case of β-CD.

The intra-unit H-bond contributions are similar to the values obtained for the most stable contribution of simple α-d-Glcp. The electrostatic interaction is the most significant term, and plays the most important role in all investigated systems (CDs, ethylene glycol and α-d-Glcp).

**Figure 7:**
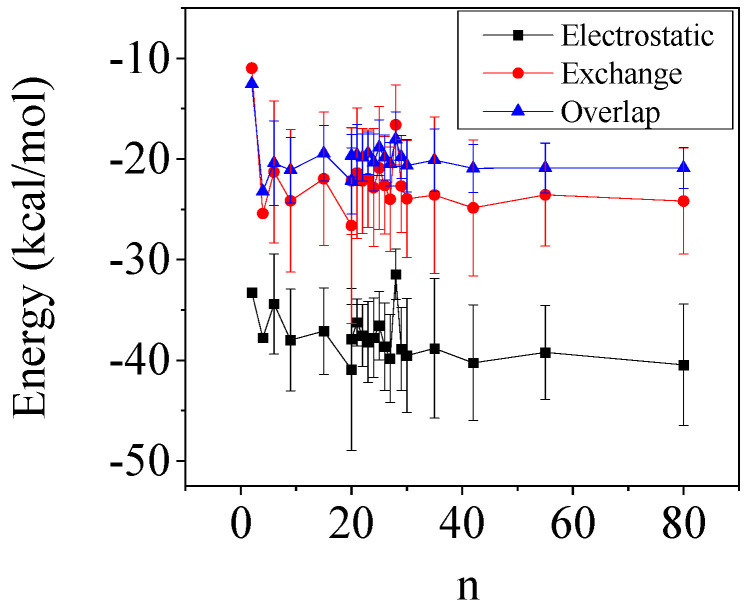
Calculated energy terms for water clusters from the CECA calculation.

We also performed similar calculation for water clusters in the range of *n =* 2–80 (Figure 7). For the studied stable water conformers, the bond order and two-body interaction energy between the H-bonded O and H atoms converges to a value of *n* > 10. The ratio of electrostatic and quantum chemistry contributions (exchange and overlapping terms) converges to a value of about 0.9. This value is significantly lower than the value obtained in the case of CD for the inter-unit bond, irrespective of whether the primary OH or the secondary OH-bond is considered. It should be noted that the strength of the O6H6_n_…O6_n−1_ H-bond (“Closed” structures) according to this analysis is in agreement with the data obtained for the water clusters.

## 3. Methods

To find the most stable structure among the several possible molecular configurations obtained from molecular mechanics calculations (OPLS_2005 force field [66,67]), we used the Maestro 12.5 program [68]. Among these tested structures, we focused on two regular structures (“Open” and “Closed”), whose presentations for β-CD are shown in Figure 2. We optimized the geometries of α-, β- and γ-CDs at the BLYP/D3, ωB97XD, and M06-2X/D3 level of theory using 6-311G** basis sets using Gaussian 09 revE software [69].

The most stable conformers of ethylene glycol (tTt, g+Gg-) [36,37], and α-d-Glcp (Tg+/cc/t, G-g+/cc/t)33, as well as one more conformer of α-d-Glcp (G-t/cc/t) [33], where the primary OH does not interact with any other oxygens of glucose, were taken from the literature. These structures were optimized using the same level of theory, but additionally, we re-optimized the obtained structure using a more flexible aug-cc-pVTZ basis set. The NBO, AIM, and CECA calculation was performed at the BLYP/6-311G** level of theory. The analyses of AIM properties were performed using the AIMALL program [70]. The LNO-CCSD(T) [71,72] calculation, which is an efficient CCSD(T) calculation, was a benchmark for energy differences of conformers. These calculations were carried out on the optimized geometries (BLYP/D3, M06-2X/D3, and ωB97XD/6-311G**) using the cc-pVTZ basis set.

The Cartesian coordinates of studied water clusters, which were well-characterized and stable conformers, were taken from the literature to get a reasonable reference for analyzing the electronic properties [64,65,71,72,73]. In these water clusters, especially in the larger sizes (*n* > 10), almost only the H-bond structures (1donor:2acceptor, 2donor:1acceptor, 2donor:2acceptor), which are the most common in liquid water, are found. Additionally, we optimized these geometries at the level of BLYP/D3/6-311G**, and we used this level of method throughout this article for analyzing the hydrogen-bonded properties. In our previous work, we have already analyzed several properties (electronic, dipole, and many-body energy decomposition) of these cluster geometries [64,65,73,74,75]. A detailed description of water clusters can be found in the Supporting Information.

Few studies have discussed Mayer’s bond order and the CECA analyzer to study H-bond interactions [45,49,75]. The principal problem with Mayer’s bond order is the calculated significant contribution to three-center bonds, especially for short bond distances and bond order between two terminal atoms. The CECA analysis can provide considerable insights into the ratio of electrostatic to chemical bonding (exchange interactions) that determine this type of interaction. For this, we investigated the correlations between bond order, electron density in BCP and energy yields from CECA analysis and delocalization energy obtained from NBO analyses for two rather large water clusters (*n =* 80,81). The obtained Pearson correlation coefficients, the correlation plots and the investigated clusters are in the Supporting Information. We found that there is a significant (>0.82) correlation between the values of the bond order, RHO(BCP), LPO…BD*OH, total, electrostatic and exchange interactions. Note that a significant correlation between OH…O distance and bond order, as well as between OH…O distance and RHO(BCP), can also be measured. This correlation is larger in the second case. The correlation coefficient between the delocalization energy (NBO) and the RHO(BCP) is also extremely high, though it is not very surprising since both quantities are from electron density. Similar correlations between these two quantities have been shown previously for different types of systems [42,76,77].

## 4. Conclusions

In this paper, we qualitatively characterized the stabilizing interactions occurring in α-, β-, and γ-CDs. For quantitative analyses, we determined the strength of the H-bond compared to standards, we used our previously studied systems: the g+Gg or tTt ethylene glycol conformers, the stable conformers of the α-d-glucose, and various water clusters.

We have demonstrated that the interactions (e.g., H-bonding and hyperconjugation) that stabilize the building blocks (α-d-glucose) of CD molecules are also present in CDs. We showed quite a good correlation between the quantum chemical descriptors from AIM, NBO, and CECA methods in those cases, where all methods detect H-bonds. This is crucial, because previous studies revealed that AIM, the most commonly used method in the literature to characterize H-bonding, is not recommended for α-d-Glcp.

Concerning inter-unit H-bonds (O6H6_n−1_…O6_n_ and O2H2_n−1_…O3_n_), which play a crucial role in stabilizing the shape of the macrocycle it was revealed that their strength decreased as the number of glucose units increased. In the most stable CDs (“Closed”) structures, the strength of the primary inter-unit H-bonds was similar, and for secondary inter-unit OH groups about half that found in the water cluster solution, respectively.

The bond order and the CECA method clearly showed the existence of a well-defined H-bonded ring along the wider rim. It could be shown that the H-bonds forming the ring in both intra- and inter-unit cases had an energy contribution characteristic of a chemical bond.

We confirmed that the AIM method for detecting the intra-unit H-bond, similar to the ethylene glycol and α-d-glucose molecules containing similar molecular details, cannot show a BCP characteristic of H-bonds along the O3H3…O2 bonds. Additionally, we detected that the delocalization energy arising from the NBO calculation cannot show any significant value for intra-unit bonds. That is, quantum chemical descriptors (RHO(BCP), delocalization energies) based on the topology of electron density failed to detect bonding interactions. At the same time, the intra-unit OH vibration showed a 40–60 cm^−1^ shift to lower frequencies compared to the non-bonded OH modes. We also showed that if we use a method operating in the Hilbert space (CECA) for the electron density analysis, then similar to ethylene glycol and α-d-glucose, all intra-unit O3H3…O2 interactions in CDs, have an exchange-like contribution. In the case of intra- and inter-unit interactions (H-bonds), this contribution is nearly 20–30% and 50% of the electrostatic interaction, respectively. However, we have shown that the RHO(BCP) values used to characterize H-bonds in water clusters show a well-defined (*p* > 0.82) correlation with both the bond order and the energy members obtained using the CECA analysis, and delocalization energy from LP(O)…BD*OH. From these data, we can conclude that for equilibrium geometries, the bond order can also be used to characterize the existence and strength of the H-bond. In some specific cases, the Hilbert space analysis (bond order, CECA) indicates the formation of a chemical bond. In these cases, however, it can be concluded that the role of electrostatic interaction is much more important than in the case of H-bonds existing in the water cluster. Our studies suggest that in the case of weak bonds (H-bonds), it is also important to investigate the extent of the contribution to the exchange interaction (chemical bonding) in these cases.

## Figures and Tables

**Table 1:** The difference of (total) energy of “Open” and “Closed” forms of CD systems (kcal/mol). (ΔEtot = E“Open” − E“Closed”). LNO-CCSD(T) values can be found in the brackets for each case.

Systems	BLYP/D3	ωB97XD	M06-2X/D3
α-CD	20.5 (18.7)	21.1 (20.8)	17.7 (16.9)
β-CD	17.6 (13.7)	15.0 (15.6)	8.4 (9.4)
γ-CD	9.0 (3.7)	7.0 (5.2)	4.3 (5.3)

**Table 2:** Intramolecular (intra- and inter-unit) OH…O distances (Å) were calculated by three different DFT methods for the two conformers (The definition of “Open” and “Closed” conformers can be found in the Methods section) of the three studied cyclodextrins. (O6H6_n_−1…O6_n_: primary inter-unit H-bond, the H-bond forming between the primary OH groups; O3H3_n_…O2_n−1_: secondary inter-unit H-bond, the H-bond forming between secondary OH groups; O2H2_n_…O3_n_: secondary intra-unit H-bond; O6H6_n−1_…O5_n_: primary inter-unit H-bond, the H-bond forming between the primary OH group and O5 ring oxygen).

Systems	H-Bond Types	BLYP/D3	ωB97XD	M06-2X/D3
α-CD “Open”	O2H2_n_…O3_n−1_	1.931	1.889	1.923
	O3H3_n_…O2_n_	2.374	2.346	2.349
	O6H6_n−1_…O5_n_	1.907	1.904	1.889
	O6H6_n−1_…O6_n_	−	−	−
α-CD “Closed”	O2H2_n_…O3_n−1_	2.245	2.175	2.158
	O3H3_n_…O2_n_	2.465	2.449	2.432
	O6H6_n−1_…O5_n_	−	−	−
	O6H6_n−1_…O6_n_	1.77	1.775	1.808
β-CD “Open”	O2H2_n_…O3_n−1_	2.072	2.051	2.056
	O3H3_n_…O2_n_	2.455	2.439	2.423
	O6H6_n−1_…O5_n_	1.991	2.016	2.051
	O6H6_n−1_…O6_n_	−	−	−
β-CD “Closed”	O2H2_n_…O3_n−1_	2.171	2.108	2.092
	O3H3_n_…O2_n_	2.465	2.451	2.434
	O6H6_n−1_…O5_n_	−	−	−
	O6H6_n−1_…O6_n_	1.789	1.787	1.811
γ-CD “Open”	O2H2_n_…O3_n−1_	2.031	2.014	2.013
	O3H3_n_…O2_n_	2.432	2.421	2.406
	O6H6_n−1_…O5_n_	1.979	2.023	2.012
	O6H6_n−1_…O6_n_	−	−	−
γ-CD “Closed”	O2H2_n_…O3_n−1_	2.184	2.114	2.103
	O3H3_n_…O2_n_	2.468	2.452	2.437
	O6H6_n−1_…O5_n_	−	−	−
	O6H6_n−1_…O6_n_	1.853	1.841	1.876

**Table 3:** Vibrational frequencies (cm^−1^) of the OH groups for the two conformers of the three investigated cyclodextrins. (The definition of “Open” and “Closed” conformers can be found in the Methods section).

Systems	Secondary Intra-Unit OH Freq. (cm^−1^)	Primary Inter-Unit OH Freq. (cm^−1^)	Secondary Inter-Unit OH Freq. (cm^−1^)
α-CD “Open”	3622–3637	3427–3440	3553–3560
α-CD “Closed”	3638–3640	3225–3283	3525–3530
β-CD “Open”	3639–3640	3440–3460	3558–3561
β-CD “Closed”	3640–3641	3305–3346	3515–3517
γ-CD “Open”	3638–3639	3486–3489	3552–3556
γ-CD “Closed”	3641–3642	3337–3366	3523–3524

**Table 4:** The vibrational frequency of the OH group for the tTt and the g+Gg- conformers of ethylene glycol.

Ethylene Glycol	O1H (cm^−1^)	O2H (cm^−1^)
tTt	3700	3701
g+Gg-	3620	3658

**Table 5:** The vibrational frequency of the OH group for the three most stable conformers [27,33] of α-d-Glcp (n: 2, 3, 4).

α-d-Glcp	O6H (cm^−1^)	O1H (cm^−1^)	OnH (cm^−1^)
G-t/cc/t	3693	3688	3654–3660
Tg+/cc/t	3603	3682	3627–3662
G-g+/cc/t	3658	3683	3627–3668

**Table 6:** Calculated average electron density at BCP (RHO(BCP)) (e/Å^3^) for different H-bond types in CDs.

Systems	O6H6_n−1_…O6_n_ Primary Inter-Unit	O6H6_n−1_…O5_n_ Primary Inter-Unit	O2H2_n_…O3_n−1_ Secondary Inter-Unit
α-CD “Open”	−	0.028	0.026
α-CD “Closed”	0.038	−	0.0132
β-CD “Open”	−	0.027	0.018
β-CD “Closed”	0.0356	−	0.0142
γ-CD “Open”	−	0.025	0.0205
γ-CD “Closed”	0.0307	−	0.015

**Table 7:** Delocalization energies obtained for inter-unit H-bonds (LPO-BD*OH) (kcal/mol). ((1) and (2) corresponding to the delocalization energy for two lone pair of oxygen atoms).

Conformers	Inter-Unit H-Bond Types	α-CD	β-CD	γ-CD
“Open”	(1) O6H6_n−1_…O5_n_	6.6	3.19	3.6
	(2) O6H6_n−1_…O5_n_	2.5	1.61	2.1
	(1) O3H3_n−1_…O2_n_	1.4	3.02	3.1
	(2) O3H3_n−1_…O2_n_	0.8	1.92	2.1
“Closed”	(1) O6H6_n−1_…O6_n_	12.6	12.81	8.3
	(2) O6H6_n−1_…O6_n_	3.6	0.0	3.3
	(1) O3H3_n−1_…O2_n_	1.5	2.1	1.8
	(2) O3H3_n−1_…O2_n_	0.9	1.14	1.2

**Table 8:** Calculated delocalization energies (kcal/mol). The O atoms are labelled as shown in Figure 1.

Type of Delocalization	α-d-Glcp Tg+/cc/t	α-CD “Open”	α-CD “Closed”	β-CD “Open”	β-CD “Closed”	γ-CD “Open”	γ-CD “Closed”
LPO1BD*C1O5		10.5	11.2	11.2	11.1	11.0	11.0
LPO5BD*C1O1	11.4	7.5	9.3	8.0	9.7	8.4	10.0
LPO1BD*C5C4		4.2	5.0	4.8	5.1	4.8	5.3
LPO5BD*C1C2	3.9	4.7	4.6	4.7	4.5	4.6	4.6
LPO5BD*C5H5	5.3	4.2	4.6	3.9	4.3	3.9	4.0
LPO5BD*C5C4	3.4	4.2	4.3	4.4	4.2	4.4	4.1

**Table 9:** Characteristic values of Mayer’s bond order and the corresponding energy contributions from the CECA method.

Systems	Type of Interactions	O…H (Å)	Bond Order	Electrostat. (kcal/mol)	Exchange (kcal/mol)	Overlap (kcal/mol)
α-CD “Open”	O6H6_n−1_…O5_n_	1.918	0.096	−23.03	−11.48	−14.62
	O2H2_n_…O3_n−1_	1.897	0.112	−26.79	−13.74	−16.82
	O3H3_n_…O2_n_	2.373	0.057	−16.13	−4.77	−7.78
α-CD “Closed”	O6H6_n−1_…O6_n_	1.770	0.141	−36.79	−19.58	−20.27
	O2H2_n_…O3_n−1_	2.242	0.078	−19.39	−7.28	−12.11
	O3H3_n_…O2_n_	2.466	0.045	−14.62	−3.58	−6.15
β-CD “Open”	O6H6_n−1_…O5_n_	1.991	0.077	−22.40	−8.79	−12.05
	O2H2_n_…O3_n−1_	2.078	0.091	−22.40	−9.54	−14.37
	O3H3_n_…O2_n_	2.455	0.046	−15.00	−3.64	−6.28
β-CD “Closed”	O6H6_n−1_…O6_n_	1.790	0.129	−35.64	−17.57	−19.08
	O2H2_n_…O3_n−1_	2.175	0.082	−20.58	−7.97	−12.93
	O3H3_n_…O2_n_	2.466	0.044	−14.75	−3.51	−6.15
γ-CD “Open”	O6H6_n−1_…O5_n_	1.978	0.079	−22.72	−9.10	−12.36
	O2H2_n_…O3_n−1_	2.031	0.093	−23.53	−10.17	−14.81
	O3H3_n_…O2_n_	2.439	0.047	−15.37	−3.76	−6.53
γ-CD “Closed”	O6H6_n−1_…O6_n_	1.854	0.125	−33.63	−15.88	−18.64
	O2H2_n_…O3_n−1_	2.179	0.079	−20.52	−7.72	−12.61
	O3H3_n_…O2_n_	2.469	0.043	−14.81	−3.39	−6.02
ethylene-glycol (g+Gg-)		2.284	0.064	−14.99	−5.71	−9.16
α-d-Glcp (Tg+/cc/t)		2.547	0.040	−15.69	−3.39	−6.34

## Data Availability

The data presented in this study are available on request from the corresponding author.

## Supplementary Materials

The following supporting information can be downloaded at: https://www.mdpi.com/article/10.3390/molecules29102205/s1, Figure S1: Water cluster with 35,42, 54,55, 80 and 81 molecules; Figure S2: The RHO(BCP) and the bond order as a function of O…H distance in water clusters; Figure S3: The bond order as a function of RHO(BCP) in water clusters; Figure S4:The delocalization energy as a function of RHO(BCP) or bond order in the water cluster; Table S1: Description of Water Clusters; Characteristic H-bonding environment of water molecules in clusters (Wan, where n is the number of water in the cluster. D: donor and A: acceptor); Table S2: Pearson correlation coefficients between the different calculated quantities for H-bonds in Wa80 and Wa81; Table S3: Electrostatic and exchange energy contributions for OH intramolecular interaction in a specified state of water molecule in the Wa80 cluster (D: donor and A: acceptor); Table S4: Electrostatic and exchange energy contributions for the OH intramolecular interaction in a specified state of water molecule in the Wa_55_ cluster (D: donor and A: acceptor); Table S5. Delocalization energies obtained for inter-unit H-bonds (LPO-BD*OH) (kcal/mol), with (1) and (2) corresponding to the delocalization energy for two lone pair of oxygen atoms using M06-2X/6-311G** and ωB97XD/6-311G** geometries; Table S6. Characteristic values of Mayer’s bond order and RHO(BCP) at the M06-2X/D3 and ωB97XD/6-311G** level of theory; Table S7: Optimized Water Cluster Coordinates for Wa_35_; Table S8: Optimized Water Cluster Coordinates for Wa_42_; Table S9: Optimized Water Cluster Coordinates for Wa_54_; Table S10: Optimized Water Cluster Coordinates for Wa_55_; Table S11: Optimized Water Cluster Coordinates for Wa_80_; Table S12: Optimized Water Cluster Coordinates for Wa_81_. Ref. [78] are cited in Supplementary Materials.
