# Supplementary material for: Systematic Study of Different Types of Interactions in α-, β- and γ-Cyclodextrin: Quantum Chemical Investigation
Source: Molecules. 2024 May 8;29(10):2205. doi: 10.3390/molecules29102205 (PMC11124371; doi:10.3390/molecules29102205)
Supplement: Supplementary file 1 [file molecules-29-02205-s001.zip › molecules-2993238-supplementary.pdf]

SUPPORTING INFORMATION

Systematic Study of Different Types of Interactions in α-, β- and γ-Cyclodextrin: Quantum Chemical Investigation

Description of Water Clusters

Analyzing the electronic properties, we retrieved a reliable reference from the literature. For the studied water clusters (n=2 to 30), the Cartesian coordinates relied on stable and well-defined conformers. [64,65,73,75] Clusters of n> 30 water molecules, each forming a cage-like structure, are in Figure S1. The coordinates of initial structures are taken from the literature [74,78]. We optimized these geometries at the level of BLYP/D3/6-311G**, and we used this level of calculation across the manuscript for analyzing the hydrogen-bonded property. In our previous work, for smaller clusters (n< 30), we have already analyzed several properties (electronic, dipole, many-body energy decomposition) of these cluster geometries [64,65,75]. The characteristic H-bonded environment of water can be described by their H-bond acceptor (A) and donor (D) number. The data for the H-bonding environment of molecule clusters n>30 are in Table S1. The coordinates of reoptimized structures are after the Figures and Tables.

Table S1. Characteristic H-bonding environment of water molecule in cluster. (Wa_n, where n is the number of water in the cluster. D: donor and A: acceptor)

	Wa_{35}	Wa_{42}	Wa_{54}	Wa_{55}	Wa_{80}	Wa_{81}
1D2A	10	12	18	15	20	22
2D1A	10	12	20	15	20	22
2D2A	15	18	24	25	40	37

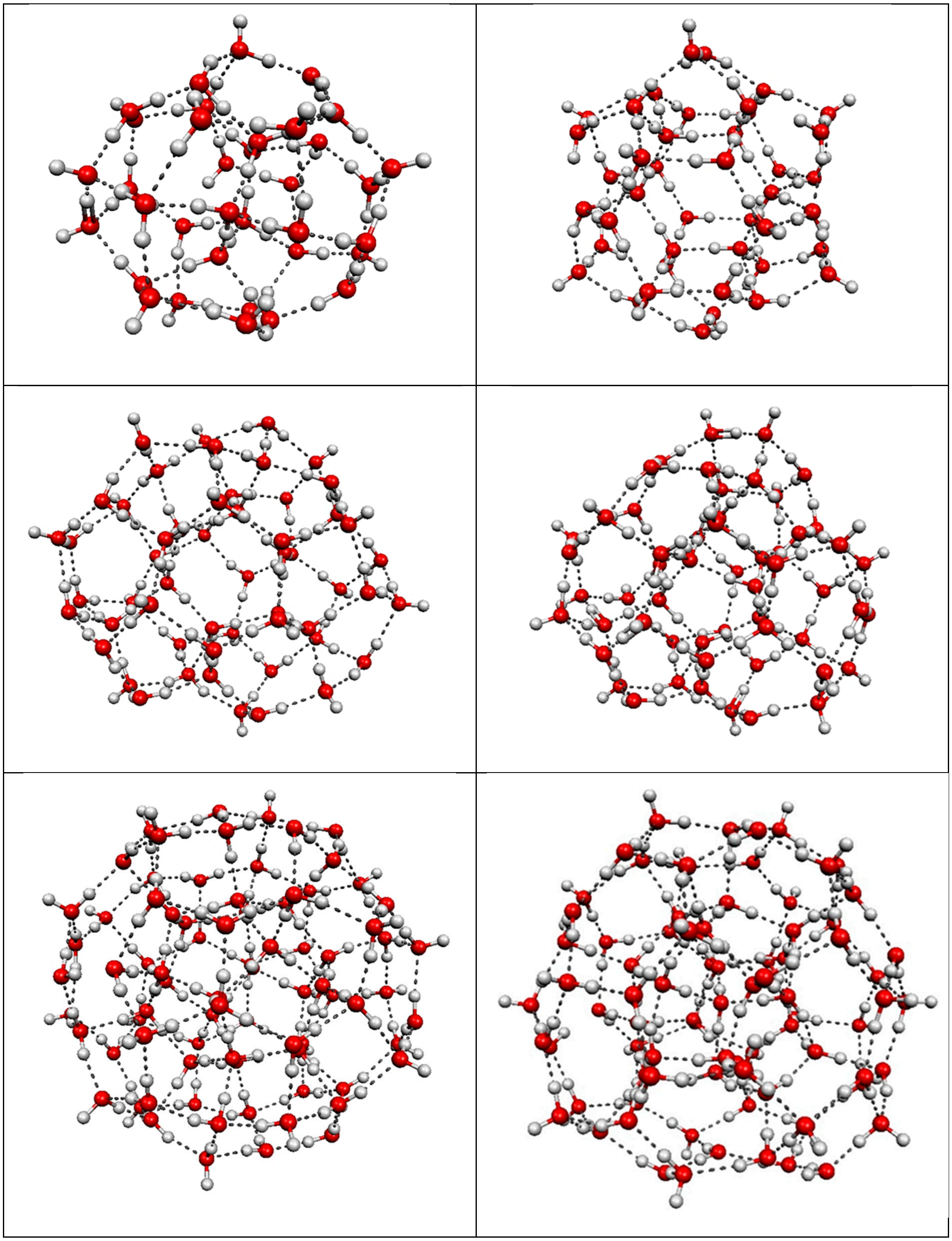

Figure S1. Water cluster with 35,42, 54,55, 80 and 81 molecules

Table S2. Pearson correlation coefficients between the different calculated quantity for H-bond in Wa_{80} and Wa_{81}

water cluster		O-H distance	BCP (e/Å)	Total	Electrostatic	Exchange
81	Bond order	-0.89	0.83	-0.95	-0.86	-0.98
	BCP (e/Å)	-0.93		0.97	0.98	0.94
80	Bond order	-0.84	0.87	-0.94	-0.81	-0.98
	BCP (e/Å)	-0.98		0.96	0.98	0.92

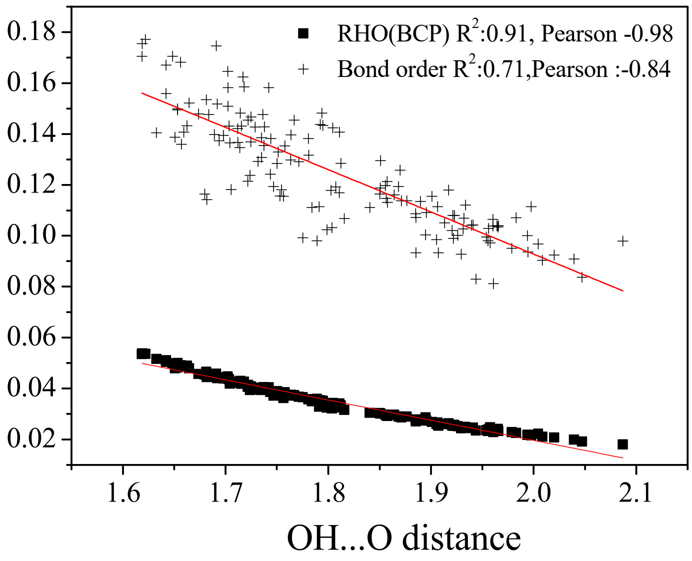

Figure S2. The RHO(BCP) and the bond order as a function of O…H distance in water clusters.

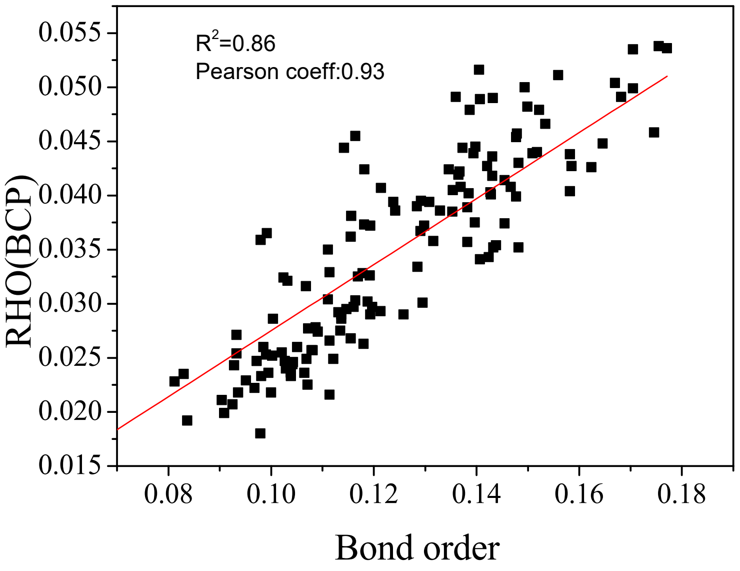

Figure S3. The bond order as a function of RHO(BCP) in water clusters.

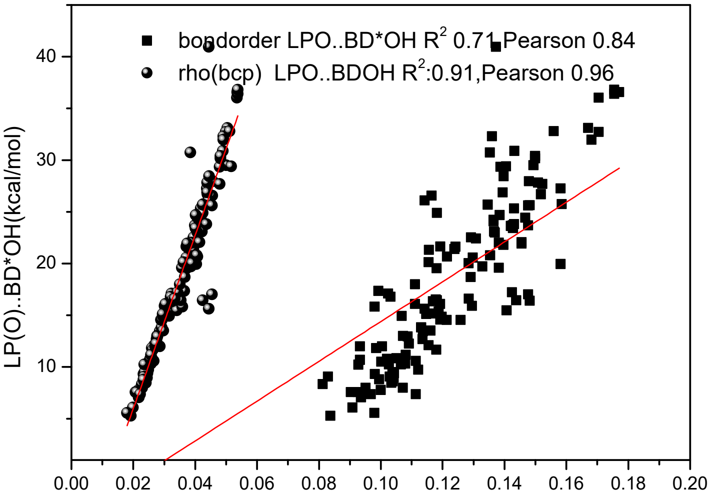

Figure S4. The delocalization energy as a function of RHO(BCP) or bond order in water cluster

Table S3. Electrostatic and exchange energy contributions for OH intramolecular interaction in a specified state of water molecule in Wa_{80} cluster (D: donor and A: acceptor).

	Electrostatic	Exchange
2D2A	-205.1	-55.1
2D1A	-219.2	-53.2
1D2A (not H-bonded hydrogen)	-234.3	-39.5
1D2A (H…in H-bond)	-200.5	-53.9

Table S4. Electrostatic and exchange energy contributions for OH intramolecular interaction in a specified state of water molecule in Wa_{55} cluster (D: donor and A: acceptor).

	Electrostatic	Exchange
2D2A	-214.2	-53.0
2D1A	-224.1	-52.8
1D2A (not H-bonded hydrogen)	-238.9	-39.8
1D2A (H…in H-bond)	-207.0	-50.97

Table S5. Delocalization energies obtained for inter-unit H bonds (LPO-BD*OH) (kcal/mol). with (1) and (2) corresponding to the delocalization energy for two lone pair of oxygen atoms using M06-2X/6-311G** and ωB97XD /6-311G** geometries.

**Conformers**	**inter-unit H bonds types**	**α-CD**	**β-CD**	**γ-CD**
"Open"	(1) $O6H6_{n-1}…O5_n$	5.6, 6.8	1.8, 2.6	2.1, 2.2
	(2) $O6H6_{n-1}…O5_n$	1.6, 1.7	2.0, 2.5	1.6, 2.0
	(1) $O3H3_{n-1}...O2_n$	7.6, 9.8	2.5, 2.6	3.1, 3.2
	(2) $O3H3_{n-1}…O2_n$	2.7, 2.8	2.6, 3.6	2.7, 3.7
"Closed"	(1) $O6H6_{n-1}…O6_n$	10.9, 17.7	12.7, 16.2	8.8, 9.8
	(2) $O6H6_{n-1}…O6_n$	4.4, 0.0	0.0, 0.0	1.4, 4.8
	(1) $O3H3_{n-1}…O2_n$	1.4, 2.5	2.5, 2.1	2.1, 2.5
	(2) $O3H3_{n-1}…O2_n$	2.0, 2.5	2.6, 2.9	2.2, 2.5

Table S6. Characteristic values of Mayer's bond order and RHO(BCP) at M06-2X/D3 and ωB97XD/6-311G** level of theory.

**Systems**	**Type of Interactions**	**RHO (BCP)**	**Bond order**
α-CD "Open"	$O6H6_{n-1}\ldots O5_n$	0.021 ,0.021	0.05, 0.05
	$O2H2_n\ldots O3_{n-1}$	0.016, 0.016	0.07, 0.07
	$O3H3_n\ldots O2_n$	no, no	0.03, 0.03
α-CD "Closed"	$O6H6_{n-1}\ldots O6_n$	0.032, 0.031	0.09, 0.10
	$O2H2_n\ldots O3_{n-1}$	0.014, 0.015	0.06, 0.07
	$O3H3_n..O2_n$	no, no	0.03, 0.3
β-CD "Open"	$O6H6_{n-1}\ldots O5_n$	0.022, 0.021	0.04, 0.05
	$O2H2_n\ldots O3_{n-1}$	0.018, 0.019	0.06, 0.07
	$O3H3_n\ldots O2_n$	no, no	0.03, 0.03
β-CD "Closed"	$O6H6_{n-1}\ldots O6_n$	0.034, 0.035	0.10, 0.10
	$O2H2_n\ldots O3_{n-1}$	0.016, 0.017	0.07, 0.07
	$O3H3_n..O2_n$	no, no	0.03, 0.03
γ-CD "Open"	$O6H6_{n-1}\ldots O5_n$	0.021, 0.021	0.04, 0.06
	$O2H2_n\ldots O3_{n-1}$	0.019, 0.019	0.07, 0.06
	$O3H3_n\ldots O2_n$	no, no	0.03, 0.03
γ-CD "Closed"	$O6H6_{n-1}\ldots O6_n$	0.027, 0.030	0.09, 0.10
	$O2H2_n\ldots O3_{n-1}$	0.019, 0.016	0.06, 0.07
	$O3H3_n..O2_n$	no, no	0.03, 0.06
ethylene-glycol (g+Gg-)		no, no	0.046, 0.043
α-d-Glcp (Tg+/cc/t)		no, no	0.028, 0.029

Table S7. Optimized Water Cluster Coordinates of Wa_{35} system (105 atoms)

O	-1.311060	3.542315	0.942372
H	-1.054432	4.212407	0.249787
H	-0.553449	2.837553	0.948345
O	-2.144552	-1.645356	-2.894618
H	-2.295252	-2.608447	-2.700919
H	-1.219236	-1.417387	-2.490879
O	-1.450737	2.700717	-2.836033
H	-2.425496	2.564583	-2.558552
H	-1.078595	3.562218	-2.494879
O	-2.944880	-2.937587	0.699042
H	-3.702234	-2.276369	0.504804
H	-2.735979	-3.530865	-0.084678
O	-2.464849	0.148955	3.384560
H	-3.254542	0.378244	2.798554
H	-2.486081	-0.820837	3.605776
O	2.591436	1.655862	-2.662680
H	2.523956	1.189641	-3.536263
H	1.684625	1.536576	-2.211558
O	1.859287	-0.343140	3.824132
H	1.066462	-0.321973	3.193644
H	1.950511	0.573641	4.181961
O	1.187582	-3.745449	1.069704
H	1.079363	-3.855416	2.057984
H	0.536340	-3.006003	0.835891
O	2.005308	-2.356331	-2.880526
H	1.991261	-1.792732	-3.696931
H	2.934014	-2.269437	-2.386455
O	2.788587	2.853488	1.463418
H	3.618107	2.209456	1.320120

H	2.556278	2.955855	2.424354
O	-0.942626	2.603754	3.629689
H	-1.518173	1.811203	3.752406
H	-1.219905	3.147625	2.856260
O	-0.770616	0.544140	-4.436143
H	-1.023258	1.411777	-4.019231
H	-1.400657	-0.173310	-4.214244
O	-2.291750	-2.608191	3.236172
H	-2.542714	-2.852724	2.282295
H	-2.639762	-3.269503	3.860894
O	-2.066099	-4.083456	-1.591827
H	-1.016059	-4.157462	-1.563706
H	-2.441032	-4.916696	-1.930726
O	-0.264729	4.666671	-1.324618
H	-0.288230	5.612101	-1.559134
H	0.727440	4.361201	-1.186988
O	1.639117	2.493729	3.985580
H	1.877097	3.004054	4.780645
H	0.625824	2.527237	3.847171
O	0.530399	-3.092390	3.580468
H	-0.415425	-2.820219	3.498740
H	1.069628	-2.356083	3.940856
O	1.718366	-0.134742	-4.611628
H	2.000467	-0.136776	-5.544220
H	0.726096	0.125734	-4.549323
O	0.456700	-4.272526	-1.519120
H	1.040029	-3.752606	-2.131662
H	0.842743	-4.362105	-0.609098
O	2.135712	3.917819	-0.969049
H	2.462071	3.696628	-0.051304

H	2.524726	3.332707	-1.659765
O	4.653089	1.232129	1.046874
H	4.514014	0.761113	-0.234406
H	5.555834	1.543476	1.241677
O	-3.541274	2.558877	0.363411
H	-2.631971	2.990496	0.648065
H	-3.735820	2.461774	-0.722504
O	4.157284	-2.038909	-1.618929
H	3.841905	-2.499958	-0.215543
H	4.947883	-2.476057	-1.984122
O	-3.965038	-0.254579	-1.924796
H	-3.213015	-0.832815	-2.383811
H	-4.358634	-0.640930	-1.042816
O	4.314002	0.376147	-1.228630
H	3.693617	0.928043	-1.814066
H	4.303173	-0.694993	-1.415361
O	-3.851914	2.207818	-2.015727
H	-3.953348	0.805706	-2.037780
H	-4.563804	2.681182	-2.481837
O	3.554084	-2.680608	0.757922
H	3.757996	-1.801668	1.552919
H	2.685982	-3.184564	0.861172
O	-4.795746	-1.177451	0.262459
H	-5.684260	-1.576697	0.220557
H	-4.694313	-0.220008	1.112335
O	3.888754	-0.858440	2.282520
H	4.258221	-0.001152	1.849261
H	3.159190	-0.700347	2.966900
O	-4.512591	0.679022	1.806961
H	-5.297424	0.933612	2.324888

H	-3.945771	1.850687	0.976272
O	-0.156623	-0.157735	2.225472
H	-1.036958	0.041791	2.674476
H	-0.269249	-0.920678	1.534902
O	0.303367	1.319319	-1.553343
H	-0.436209	1.824170	-2.026827
H	0.393800	1.543741	-0.546682
O	0.548416	1.828244	0.874893
H	1.485897	2.205843	1.074866
H	0.305306	1.017981	1.466980
O	-0.542848	-1.902522	0.488382
H	-1.483719	-2.240615	0.564867
H	-0.294927	-1.593798	-0.472897
O	0.070943	-1.139879	-1.797390
H	0.207293	-0.114108	-1.744417
H	0.886781	-1.642471	-2.178843

Table S8. Optimized Water Cluster Coordinates of Wa_{42} system (126 atoms)

O	1.854442	3.707521	2.125969
H	1.568427	3.179741	1.349009
H	1.311487	4.507806	2.060163
O	-4.141715	-0.2505500	2.120729
H	-4.563454	-1.120717	2.053361
H	-3.540990	-0.2335950	1.344255
O	2.284651	-3.463845	2.120021
H	3.249210	-3.394083	2.053998
H	1.970805	-2.950747	1.343984
O	-2.033272	-3.666735	2.250788
H	-1.833125	-3.220486	3.096833
H	-2.984411	-3.559505	2.073988

O	4.190262	7.0592001E-02	2.256931
H	3.702634	1.9966001E-02	3.102284
H	4.572673	-0.8067540	2.079541
O	-2.159073	3.590635	2.257471
H	-1.872537	3.192675	3.102755
H	-1.590437	4.360847	2.081954
O	-1.854030	-3.707397	-2.125693
H	-1.311020	-4.507652	-2.060097
H	-1.568009	-3.179653	-1.348717
O	-2.284779	3.463483	-2.119729
H	-1.970935	2.950351	-1.343716
H	-3.249338	3.393658	-2.053883
O	4.141426	0.2508030	-2.120407
H	4.563096	1.121013	-2.053255
H	3.540749	0.2338810	-1.343903
O	2.033512	3.666860	-2.250724
H	1.833660	3.221293	-3.097145
H	2.984605	3.559632	-2.073653
O	2.159086	-3.590899	-2.257385
H	1.590487	-4.361093	-2.081633
H	1.873019	-3.193527	-3.103057
O	-4.190515	-7.0436001E-02	-2.256848
H	-3.703603	-1.9896001E-02	-3.102568
H	-4.572913	0.8068670	-2.079194
O	-4.545440	2.422202	1.221606
H	-3.838650	2.927994	1.651046
H	-4.591883	1.557558	1.657237
O	0.1729850	-5.147729	1.215986
H	-0.6189780	-4.789150	1.644885
H	0.9444780	-4.756274	1.653188

O	4.370910	2.723389	1.225038
H	4.455842	1.857806	1.653131
H	3.645279	3.194990	1.661616
O	4.849346	-2.510119	1.409516
H	4.712760	-2.467493	0.4273540
H	5.709372	-2.915288	1.529186
O	-0.2527690	5.453135	1.415012
H	-0.2199350	5.314463	0.4327720
H	-0.3314010	6.400439	1.535627
O	-4.598507	-2.947416	1.405699
H	-5.379154	-3.490129	1.524726
H	-4.493383	-2.848741	0.4237320
O	-4.849636	2.509965	-1.409352
H	-5.709471	2.915463	-1.529253
H	-4.713098	2.467540	-0.4271660
O	4.598497	2.947709	-1.405565
H	4.493598	2.849002	-0.4235650
H	5.379345	3.490072	-1.524851
O	0.2530510	-5.453295	-1.414897
H	0.3312850	-6.400599	-1.535743
H	0.2200780	-5.314764	-0.4326310
O	4.545397	-2.421920	-1.221570
H	3.838663	-2.927737	-1.651056
H	4.591656	-1.557189	-1.657048
O	-4.370595	-2.723468	-1.225021
H	-4.455592	-1.857926	-1.653163
H	-3.644809	-3.194960	-1.661453
O	-0.1731990	5.147528	-1.215990
H	-0.9446920	4.755889	-1.653035
H	0.6187560	4.788999	-1.644931

O	1.268744	2.397792	-4.557137
H	0.2653280	2.317310	-4.485825
H	1.435665	2.877529	-5.369822
O	1.335771	2.252941	4.401531
H	1.539975	2.811498	3.628128
H	0.3674370	2.232208	4.483062
O	-2.704764	-9.5077999E-02	-4.560693
H	-2.133128	-0.9236460	-4.489489
H	-3.201916	-0.1894740	-5.374553
O	-2.625464	2.5508000E-02	4.398559
H	-3.209774	-7.6491997E-02	3.623936
H	-2.123428	-0.8027180	4.480712
O	1.441174	-2.289725	-4.560993
H	1.873308	-1.380651	-4.488848
H	1.771958	-2.672777	-5.374831
O	1.284496	-2.291169	4.397727
H	1.666146	-2.745896	3.623502
H	1.750762	-1.442366	4.480886
O	-1.335494	-2.252847	-4.401558
H	-1.539699	-2.811331	-3.628121
H	-0.3672110	-2.232573	-4.483408
O	-1.268868	-2.396884	4.556488
H	-0.2652490	-2.317044	4.485469
H	-1.436327	-2.876908	5.368894
O	2.625281	-2.5340000E-02	-4.398512
H	2.123602	0.8030290	-4.480903
H	3.209451	7.6572001E-02	-3.623785
O	2.703976	9.4567999E-02	4.559980
H	3.201616	0.1884820	5.373599
H	2.132930	0.9237580	4.489114

O	-1.284625	2.291029	-4.397760
H	-1.751264	1.442510	-4.481269
H	-1.666245	2.745728	-3.623519
O	-1.440243	2.289184	4.560204
H	-1.771019	2.672714	5.373826
H	-1.873030	1.380257	4.488190
O	-1.432999	2.173918	7.5855002E-02
H	-1.705611	2.644669	0.8912370
H	-0.4442000	2.191684	4.0206000E-02
O	1.166171	2.327076	-7.2003998E-02
H	1.438772	2.799300	-0.8864970
H	1.675842	1.479558	-3.6509998E-02
O	-1.165967	-2.326762	7.2269998E-02
H	-1.438702	-2.799169	0.8866370
H	-1.675924	-1.479422	3.6765002E-02
O	-2.599917	-0.1534430	-7.5429000E-02
H	-3.143645	-0.1527150	-0.8909690
H	-2.120672	0.7115900	-3.8488001E-02
O	2.599531	0.1534860	7.5723000E-02
H	3.143461	0.1527470	0.8911530
H	2.120588	-0.7117040	3.8757000E-02
O	1.433164	-2.174240	-7.5594999E-02
H	0.4443480	-2.191680	-3.9976001E-02
H	1.705692	-2.644788	-0.8911020

Table S9. Optimized Water Cluster Coordinates of Wa_{54} system (162 atoms)

O	-6.256077	-1.295262	1.588510
H	-6.388185	-0.332659	1.508024
H	-5.743202	-1.561721	0.797448
O	-4.700610	-2.051689	-0.628409

H	-3.921223	-1.457234	-0.675931
H	-5.277101	-1.808512	-1.406879
O	-4.843865	-2.416559	3.633417
H	-5.417433	-2.010902	2.938476
H	-4.435275	-3.223273	3.234918
O	-6.182919	-1.250403	-2.681501
H	-6.342132	-0.308577	-2.442787
H	-5.593762	-1.280643	-3.454645
O	-6.638865	1.544256	1.161605
H	-7.503899	1.790393	1.512606
H	-5.971554	2.080710	1.670871
O	-3.987611	-4.603328	-0.423985
H	-4.305585	-3.659858	-0.527148
H	-4.684467	-5.172574	-0.773427
O	-6.466306	1.348992	-1.688377
H	-5.732977	1.949920	-1.903973
H	-6.570637	1.395636	-0.715785
O	-3.551189	-4.547417	2.442685
H	-2.586358	-4.378078	2.431072
H	-3.786133	-4.683292	1.507133
O	-3.114307	-0.801236	4.543852
H	-3.424352	-0.584086	5.432549
H	-3.824358	-1.444491	4.155940
O	-3.937244	-1.509874	-4.576566
H	-3.530943	-0.670161	-4.247156
H	-4.108932	-1.399580	-5.520002
O	-4.849217	2.928842	2.611833
H	-4.461292	3.779387	2.334977
H	-4.084771	2.392557	2.917754
O	-1.520062	-4.396303	-1.630111

H	-2.365275	-4.626005	-1.177513
H	-0.873407	-5.125885	-1.503915
O	-0.840787	-3.771852	2.239620
H	-0.145811	-4.450052	2.045729
H	-0.801252	-3.146727	1.486958
O	-4.216604	2.892588	-2.715005
H	-3.710468	2.084108	-3.000523
H	-4.621450	3.271158	-3.505265
O	-2.518299	1.493613	3.173053
H	-1.862835	1.924633	3.767168
H	-2.734156	0.621623	3.592442
O	-1.773745	-3.444874	-4.202326
H	-1.784328	-3.814121	-3.288836
H	-2.526859	-2.830394	-4.290306
O	-2.824851	0.658398	-3.361961
H	-1.985494	0.913416	-3.802391
H	-2.591061	0.226513	-2.486862
O	-0.869362	-2.454743	4.650297
H	-0.870356	-2.968327	3.808325
H	-1.662979	-1.875743	4.636287
O	0.710482	-5.997764	-1.311886
H	0.796786	-6.925684	-1.563099
H	1.329182	-5.478795	-1.908294
O	-3.308911	5.188365	1.715637
H	-3.639764	6.092582	1.783105
H	-3.202047	4.985034	0.755745
O	1.108374	-5.532997	1.497091
H	2.023338	-5.256465	1.697031
H	1.059215	-5.724764	0.540736
O	-2.760859	4.267453	-0.775919

H	-3.336466	3.893987	-1.479097
H	-2.029222	4.738334	-1.225117
O	0.745370	-3.049534	-4.832848
H	0.886776	-3.540054	-5.653018
H	-0.231018	-3.155419	-4.615456
O	-0.637273	2.812746	4.743865
H	0.220221	2.291510	4.849905
H	-0.898620	3.070020	5.636990
O	-0.571711	1.554819	-4.709136
H	-0.783470	2.007377	-5.535315
H	-0.169760	2.233663	-4.100815
O	1.413987	-1.358282	4.954335
H	1.806494	-1.755806	5.742132
H	0.482467	-1.766704	4.860552
O	2.265807	-4.560088	-2.883814
H	2.936941	-4.009936	-2.420949
H	1.783648	-3.982455	-3.507137
O	-0.623204	4.928786	2.894965
H	-1.529461	4.999250	2.543135
H	-0.631749	4.229215	3.581990
O	1.497833	-0.394891	-5.036843
H	1.224237	-1.338287	-4.962555
H	0.693719	0.152358	-4.968602
O	1.531972	1.413602	5.139401
H	1.452844	0.436191	5.071583
H	2.422865	1.644552	4.801234
O	3.659449	-4.507412	2.347847
H	3.898673	-4.958022	3.167554
H	3.362585	-3.586522	2.596654
O	-0.639256	5.614394	-2.212809

H	-0.949011	6.246169	-2.873802
H	-0.229620	4.851817	-2.691925
O	2.914677	-1.976319	2.720358
H	3.726841	-1.435348	2.941503
H	2.294107	-1.794950	3.466063
O	0.546473	3.333841	-3.035135
H	1.477533	3.381474	-3.345419
H	0.557392	3.009549	-2.085552
O	4.027959	-2.929671	-1.469417
H	4.667813	-3.290018	-0.792503
H	4.540402	-2.291397	-2.005211
O	1.423629	4.449009	1.209138
H	0.687379	4.685053	1.834776
H	1.593510	5.230090	0.631544
O	5.658958	-3.700145	0.503864
H	6.092124	-2.901729	0.867330
H	5.081602	-4.063972	1.206121
O	1.898454	6.246330	-0.802786
H	2.614134	5.790867	-1.283344
H	1.085949	6.164164	-1.332598
O	3.321726	0.674824	-3.457981
H	2.971047	0.674333	-2.522603
H	2.644007	0.204333	-4.027956
O	4.133248	1.971082	4.195192
H	4.049596	2.624860	3.433192
H	4.664561	2.409407	4.871859
O	5.061502	-0.586646	3.413135
H	4.860554	0.349216	3.620932
H	5.762311	-0.631467	2.732520
O	3.176331	3.221348	-4.035576

H	3.313991	2.231169	-3.877290
H	3.328690	3.383075	-4.975117
O	5.444871	-0.908756	-2.919475
H	4.720642	-0.317370	-3.251045
H	5.976837	-1.171606	-3.680824
O	3.934309	3.618138	2.181283
H	4.289507	3.196194	1.371035
H	3.037470	3.939673	1.942472
O	6.870276	-1.215393	1.347678
H	6.824375	-0.583782	0.572539
H	7.804520	-1.292958	1.578365
O	4.134449	4.703182	-1.947633
H	3.926166	4.257193	-2.800455
H	4.911968	5.257573	-2.093620
O	6.696283	0.451300	-0.714100
H	6.334238	-0.000509	-1.500957
H	6.107588	1.217836	-0.545395
O	4.722730	2.414902	-0.244825
H	3.954279	1.817796	-0.417898
H	4.602477	3.188049	-0.829823
O	-2.399654	-0.400318	-0.960702
H	-2.295504	0.374887	-0.360011
H	-1.669263	-1.028165	-0.717193
O	-0.574554	-2.242186	-0.180501
H	0.396212	-2.079954	-0.188054
H	-0.777671	-3.020695	-0.750663
O	-1.876010	1.833796	0.541122
H	-2.075427	1.692052	1.503851
H	-2.319570	2.654514	0.251208
O	2.149359	-1.630800	0.086068

H	2.385277	-1.739327	1.041369
H	2.804099	-2.154732	-0.438011
O	0.564951	2.558783	-0.472537
H	-0.271558	2.210085	-0.076652
H	0.888985	3.260135	0.166387
O	2.580753	0.820401	-0.894763
H	2.433423	-0.066004	-0.462584
H	1.791042	1.377675	-0.682164

Table S10. Optimized Water Cluster Coordinates of Wa_{55} system (165 atoms)

O	-6.009526	-1.235886	1.658474
H	-5.903294	-0.137245	1.677115
H	-6.912981	-1.543091	1.844156
O	-4.629116	-1.697839	-0.467805
H	-3.637697	-1.209014	-0.322036
H	-5.194977	-1.630335	0.381659
O	-4.191809	-2.568408	3.083619
H	-4.909684	-2.066135	2.613960
H	-3.832202	-3.394384	2.560975
O	-5.840185	-0.539980	-2.258686
H	-5.293447	-1.103880	-1.455804
H	-6.722214	-0.920555	-2.426573
O	-5.708647	1.168912	1.538085
H	-5.791254	1.528752	0.615808
H	-5.088881	1.672793	2.154119
O	-4.050982	-4.211063	-0.905179
H	-4.293108	-3.224887	-0.776521
H	-4.799363	-4.724275	-1.255579
O	-5.978607	1.807329	-1.051944
H	-5.918393	0.950698	-1.592991

H	-6.705697	2.377010	-1.357200
O	-3.229984	-4.454494	1.723753
H	-2.219252	-4.422375	1.702432
H	-3.579750	-4.561544	0.808982
O	-2.630931	-1.354108	4.452816
H	-2.785631	-1.462123	5.408248
H	-3.408762	-1.932403	3.816869
O	-4.059761	-0.705459	-4.151898
H	-4.850060	-0.655034	-3.517012
H	-4.232206	-0.275463	-5.007484
O	-4.106711	2.374955	3.205233
H	-4.565219	2.739272	3.982749
H	-3.256221	1.805292	3.478083
O	-1.728405	-4.203501	-2.243311
H	-2.546802	-4.411085	-1.714845
H	-0.949905	-4.863219	-2.205649
O	-0.678422	-3.981252	1.500387
H	0.067867	-4.634445	1.226023
H	-0.703943	-3.282705	0.778440
O	-3.517522	3.046761	-1.537811
H	-3.054967	2.280485	-1.986804
H	-4.416434	2.750929	-1.271049
O	-2.063360	1.032578	3.731541
H	-1.317239	1.429936	4.303202
H	-2.272982	0.055276	4.004498
O	-2.048301	-2.611581	-4.296750
H	-2.000708	-3.273281	-3.540703
H	-2.796110	-1.971855	-4.233233
O	-2.532467	0.789036	-2.492913
H	-3.034269	0.268402	-3.173205

H	-2.375653	0.235093	-1.667278
O	-0.447868	-2.753884	3.797913
H	-0.562224	-3.297534	2.955692
H	-1.267772	-2.255925	4.072594
O	0.535542	-5.398331	-2.148524
H	0.712247	-6.281635	-2.517722
H	1.077111	-4.586752	-2.702207
O	-2.540291	4.328803	2.128117
H	-3.232607	3.692264	2.430649
H	-2.448391	4.630103	0.901427
O	1.247324	-5.302865	0.484610
H	2.145906	-4.954955	0.711325
H	1.063795	-5.348634	-0.495311
O	-2.358010	4.835994	-0.214964
H	-2.805590	4.132374	-0.829821
H	-1.576975	5.369329	-0.574705
O	0.270795	-2.163938	-5.081708
H	0.423986	-2.513972	-5.977897
H	-0.735896	-2.293750	-4.793620
O	-0.100542	2.028838	5.112535
H	0.825220	1.453156	5.064154
H	-0.282779	2.308741	6.026748
O	-0.909320	2.045470	-4.195703
H	-1.410699	1.532867	-3.508306
H	-0.626210	2.903031	-3.784475
O	1.685014	-1.561527	3.773634
H	2.469127	-2.170823	3.690367
H	0.755181	-2.106582	3.861178
O	1.661691	-3.579846	-3.292459
H	2.448378	-3.135256	-2.832224

H	1.189284	-3.009460	-3.967339
O	-0.375416	4.081665	3.345644
H	-1.665827	4.269161	2.697261
H	-0.219894	3.393156	4.042015
O	0.853163	0.524773	-5.321469
H	0.641367	-0.439833	-5.227544
H	0.097763	1.128853	-5.002184
O	1.938212	0.665241	5.038486
H	1.823861	-0.274008	4.664266
H	2.836332	1.008726	4.790232
O	3.816055	-4.601077	1.304606
H	4.055947	-5.535632	1.444477
H	4.472416	-4.108473	0.545731
O	-0.302869	6.122391	-1.180552
H	0.707646	6.086734	-0.635718
H	-0.529857	7.010282	-1.508484
O	3.812768	-3.190790	3.440354
H	3.767933	-3.774927	2.583175
H	4.004828	-3.727518	4.228609
O	0.109645	3.994741	-2.628626
H	1.035857	3.970816	-3.012538
H	-0.118147	4.906139	-2.266438
O	3.525944	-2.368992	-1.961908
H	4.213613	-2.879995	-1.417558
H	3.953082	-1.570985	-2.481467
O	1.371282	3.930033	1.416971
H	0.353507	4.094351	2.651039
H	2.098064	3.283671	1.652381
O	5.233833	-3.435860	-0.319523
H	5.634769	-2.180693	0.166295

H	5.965664	-3.948870	-0.707610
O	1.853358	5.919349	-0.053146
H	1.655788	4.793211	0.911112
H	2.213422	6.728847	0.352778
O	2.669497	1.363650	-3.849109
H	2.242025	1.218795	-2.938917
H	1.998946	0.972432	-4.576781
O	4.291781	1.338611	3.898355
H	3.825176	1.670741	3.013635
H	4.919884	1.984074	4.265775
O	5.213653	-0.973042	2.918982
H	4.922195	-0.128677	3.365479
H	4.729299	-1.787061	3.224597
O	2.550070	3.855778	-3.779844
H	2.692469	2.805217	-3.899634
H	2.622328	4.333240	-4.624159
O	4.566773	-0.355425	-3.085474
H	3.957911	0.310196	-3.526307
H	5.206843	0.078471	-2.468804
O	3.046264	1.876541	1.807799
H	3.539692	1.993162	0.920928
H	2.371744	1.103343	1.743878
O	5.862415	-1.215980	0.567483
H	5.947878	-0.422174	-0.064372
H	5.618063	-1.046051	1.596590
O	3.572415	4.482234	-1.411159
H	3.298953	4.389938	-2.366380
H	2.953149	5.101409	-0.899524
O	5.962082	0.794943	-1.020680
H	6.806705	1.249961	-1.178494

H	5.149068	1.468642	-0.824153
O	3.964754	2.183073	-0.584272
H	3.162215	1.674662	-0.970202
H	3.876297	3.214294	-0.888029
O	-2.405559	-0.678037	-0.300648
H	-2.029634	-0.034129	0.408796
H	-1.667008	-1.415666	-0.460835
O	-0.670093	-2.413660	-0.653131
H	0.295370	-2.053953	-0.735083
H	-0.974181	-3.060096	-1.362241
O	-1.110575	0.880025	1.246337
H	-1.469165	1.087618	2.162857
H	-0.848758	1.661670	0.654452
O	1.632726	-1.392980	-0.581898
H	1.711918	-1.045813	0.364907
H	2.450318	-1.834937	-1.030657
O	0.014890	2.605781	-0.349030
H	-0.146459	3.114507	-1.205372
H	0.512276	3.181429	0.336021
O	1.803818	1.044584	-1.429224
H	1.678655	0.063736	-1.206785
H	1.066783	1.607673	-1.016306
O	1.383164	-0.125141	1.681025
H	0.433784	0.180099	1.544292
H	1.498265	-0.708091	2.527849

Table S11. Optimized Water Cluster Coordinates of Wa_{80} system (240 atoms)

O	-3.579181	6.143738	-0.523887
H	-3.957282	7.004875	-0.742978
H	-3.667171	5.580809	-1.343992
O	-4.413995	4.607244	1.660248
H	-4.214442	5.250972	0.943392
H	-3.981719	4.934185	2.484463
O	-3.662804	4.488034	-2.647314
H	-4.499178	4.089572	-2.965097
H	-3.156439	3.721054	-2.267876
O	-6.452157	2.716187	1.365269
H	-5.741537	3.387094	1.501245
H	-7.276784	3.166045	1.591607
O	-6.515174	0.219958	2.940423
H	-6.440462	1.058108	2.446027
H	-5.881098	0.294695	3.686950
O	-2.865885	5.210254	3.880654
H	-3.073196	5.900569	4.523487
H	-1.944450	5.409126	3.540656
O	-2.790356	2.802535	5.625743
H	-2.845003	3.533055	4.984268
H	-3.409665	2.103257	5.340044
O	-4.322396	0.477460	4.696508
H	-4.036051	-0.258969	5.272127
H	-3.793862	0.388223	3.865907
O	0.801225	5.133715	-3.690485
H	1.666448	4.869913	-4.087808
H	0.982210	5.837338	-3.029514
O	1.266763	6.901271	-1.544154
H	1.316062	7.848289	-1.727668

H	0.527883	6.802307	-0.880769
O	-1.777496	4.657162	-4.912967
H	-2.412086	4.741076	-4.177349
H	-0.907665	4.938389	-4.569173
O	-0.764044	6.791903	0.213718
H	-0.620085	6.459506	1.128100
H	-1.660240	6.515889	-0.050286
O	-0.461857	5.596924	2.754268
H	-0.290107	4.682060	2.410988
H	0.368288	5.763912	3.274299
O	3.951039	6.169971	-0.634528
H	3.038136	6.379630	-0.913538
H	4.282255	5.504974	-1.264658
O	3.604701	4.657867	1.662820
H	2.974653	4.138136	1.114095
H	3.890122	5.347689	1.019588
O	1.967495	5.480811	3.947337
H	1.958773	4.680602	4.512706
H	2.586737	5.286695	3.215107
O	0.531261	0.838490	-6.089289
H	0.420721	-0.101801	-6.395902
H	-0.341497	1.314997	-6.179530
O	3.075268	1.601832	-6.073604
H	3.374452	1.837662	-6.961404
H	2.102688	1.382967	-6.162297
O	-1.836194	2.104762	-6.021102
H	-1.813840	3.026216	-5.669952
H	-2.571262	1.656760	-5.563574
O	3.210532	4.137641	-4.594431
H	3.176938	3.268955	-5.036949

H	3.877832	4.075595	-3.884783
O	5.196075	4.234731	-2.487500
H	5.542710	3.416200	-2.065726
H	5.968929	4.721647	-2.801196
O	4.274875	-0.583274	-4.771137
H	3.877854	-1.408525	-5.151138
H	3.916733	0.178317	-5.285235
O	6.449608	-0.432452	-3.176829
H	5.684388	-0.496974	-3.811047
H	7.234445	-0.325686	-3.730262
O	6.088483	1.837972	-1.359913
H	6.558963	1.686948	-0.505833
H	6.272471	1.081352	-1.950733
O	-4.587568	-1.717906	-5.339592
H	-5.179847	-1.800424	-6.097314
H	-4.494181	-0.745959	-5.155735
O	-1.880871	-2.829083	-5.364116
H	-2.782246	-2.523670	-5.579955
H	-1.627218	-2.287009	-4.576451
O	-4.129506	0.836170	-4.545352
H	-4.799714	1.529761	-4.381877
H	-3.831878	0.570930	-3.640528
O	0.419221	-1.787197	-6.701739
H	-0.384941	-2.240226	-6.370783
H	1.190110	-2.231302	-6.301275
O	2.987599	-2.827575	-5.709774
H	2.919978	-3.587691	-5.047230
H	3.341237	-3.229316	-6.514475
O	-1.971570	-5.486430	-4.244411
H	-1.946563	-4.620377	-4.703240

H	-2.653235	-5.401401	-3.554991
O	0.468541	-5.678225	-2.909922
H	-0.312006	-5.855007	-3.482595
H	0.215101	-4.829169	-2.475861
O	2.889050	-4.868407	-4.071348
H	2.026090	-5.217057	-3.745294
H	3.487663	-4.846025	-3.299607
O	-6.798941	1.590270	-1.279071
H	-6.615373	1.939933	-0.384969
H	-6.778899	0.614209	-1.231087
O	-6.946067	-1.320186	-1.188195
H	-6.743583	-1.603518	-0.260577
H	-7.877212	-1.530762	-1.336253
O	-5.868902	2.846084	-3.448719
H	-6.217563	2.384866	-2.635081
H	-6.639334	3.201662	-3.908904
O	-5.447895	-3.084534	-2.961122
H	-5.142965	-2.602846	-3.757101
H	-5.884883	-2.436511	-2.375796
O	-4.171800	-5.311478	-2.177845
H	-4.755033	-6.003815	-2.515455
H	-4.620030	-4.453007	-2.419496
O	-6.113522	-1.882469	1.318369
H	-5.910987	-2.716351	1.788599
H	-6.286824	-1.158148	1.984103
O	-5.099349	-4.328328	2.442014
H	-4.567645	-4.768956	1.733267
H	-5.739265	-4.987331	2.741193
O	-3.474538	-5.413735	0.508996
H	-3.711748	-5.502424	-0.445218

H	-2.780762	-6.066071	0.749940
O	3.686589	-6.045140	0.378899
H	3.681317	-5.651823	1.269568
H	2.770199	-6.353357	0.186332
O	1.106731	-6.804709	-0.355094
H	0.317554	-6.690460	0.201138
H	0.853034	-6.557632	-1.269553
O	-1.235933	-6.739769	1.544118
H	-1.207132	-7.665094	1.819427
H	-1.039265	-6.200532	2.356377
O	3.651211	-4.488104	2.891187
H	3.067144	-4.671570	3.656657
H	4.436641	-3.999383	3.217098
O	6.776564	-1.515290	1.420535
H	6.630199	-1.848754	0.515591
H	6.765320	-0.536815	1.375829
O	6.613082	-2.572479	-1.309683
H	6.534887	-1.861189	-1.984706
H	7.473725	-2.985732	-1.459514
O	4.371633	-4.464881	-1.653496
H	4.230678	-5.092843	-0.887799
H	5.201388	-3.975552	-1.509601
O	5.798514	-2.762317	3.581193
H	6.131681	-2.307446	2.756956
H	6.581451	-3.128241	4.011760
O	4.607169	1.875819	5.291992
H	4.494531	0.896965	5.197319
H	5.242278	2.018103	6.004707
O	5.454100	2.862811	2.700630
H	4.828224	3.535754	2.339055

H	5.129378	2.579875	3.577498
O	6.957420	1.343581	1.207391
H	6.339108	1.901848	1.782805
H	7.849395	1.604055	1.471701
O	4.069524	-0.736144	4.660398
H	3.785362	-0.475435	3.749634
H	4.755543	-1.414999	4.502274
O	-0.634696	-0.757691	6.314435
H	0.237296	-1.217131	6.360299
H	-0.504354	0.200338	6.579345
O	-0.432943	1.849573	6.805833
H	-1.222018	2.278414	6.405958
H	0.355101	2.312809	6.461207
O	1.870255	2.959434	5.370221
H	1.618474	2.345336	4.640123
H	2.782728	2.696268	5.589387
O	1.814457	-2.052945	6.063565
H	1.782333	-2.947635	5.678903
H	2.545305	-1.581475	5.618118
O	-0.725419	-5.081379	3.673309
H	-0.523186	-4.240258	3.175771
H	-1.569888	-4.862732	4.141590
O	-3.134691	-4.185221	4.667996
H	-3.147553	-3.348464	5.176197
H	-3.811434	-4.112477	3.970578
O	-3.131001	-1.745207	6.211991
H	-2.199498	-1.428027	6.361224
H	-3.504097	-1.897521	7.089762
O	1.696900	-4.799644	4.960728
H	0.799412	-4.985215	4.580507

H	1.828143	-5.442121	5.669594
O	2.812401	-0.398914	-2.422627
H	3.402177	-0.470263	-3.205470
H	1.963650	-0.017021	-2.793156
O	1.896959	3.171213	-0.011550
H	2.435294	2.347521	-0.039223
H	1.159596	3.065668	0.643821
O	-1.153527	-1.397354	-3.171280
H	-0.731024	-2.100432	-2.608188
H	-0.494727	-0.666297	-3.264467
O	-2.252167	2.504051	-1.525355
H	-1.299426	2.667502	-1.760075
H	-2.497737	1.582061	-1.800759
O	2.286192	-2.575775	1.526565
H	2.817018	-3.292495	1.953173
H	2.385133	-2.652220	0.538820
O	1.141906	1.288807	3.334570
H	1.884560	0.832966	2.867868
H	0.470415	0.579446	3.505846
O	-1.913555	-3.153298	0.333175
H	-2.431397	-3.982147	0.446288
H	-1.329695	-3.038234	1.132226
O	-2.890902	0.346096	2.402899
H	-2.788672	1.231010	1.967084
H	-3.187999	-0.242369	1.663528
O	-2.583614	2.639286	1.091838
H	-3.241431	3.344066	1.289654
H	-2.503398	2.576177	0.099931
O	-3.681498	-1.127538	0.281903
H	-3.059685	-1.900344	0.263964

H	-4.558086	-1.457596	0.585042
O	-0.724604	-0.594517	3.647944
H	-1.551692	-0.220280	3.247648
H	-0.809889	-0.655104	4.636288
O	-0.110286	-3.322568	-1.678691
H	0.806945	-3.106024	-1.376576
H	-0.722709	-3.328463	-0.904061
O	3.142748	-0.121298	2.178143
H	3.480119	0.304692	1.357855
H	2.820336	-1.020357	1.909992
O	0.294948	2.977894	-2.182397
H	0.435337	3.773542	-2.763482
H	0.919833	3.088297	-1.418932
O	-0.044986	3.111794	1.807321
H	0.319058	2.431864	2.432425
H	-0.972648	2.870457	1.568054
O	-3.124602	0.045900	-2.147030
H	-2.412849	-0.574785	-2.463596
H	-3.489914	-0.349469	-1.322145
O	-0.229871	-2.835051	2.351035
H	0.703121	-2.749457	2.018257
H	-0.412305	-2.012522	2.885901
O	2.428017	-2.698384	-1.104089
H	3.124086	-3.353787	-1.373079
H	2.594783	-1.862413	-1.609643
O	3.612586	1.039023	-0.278132
H	4.458437	1.424268	-0.587616
H	3.307615	0.493398	-1.045690
O	0.618374	0.629407	-3.423164
H	0.612867	0.750502	-4.411015

H	0.484463	1.521861	-3.006325

Table S12. Optimized Water Cluster Coordinates of Wa_{81} system (243 atoms)

O	3.149012	-0.612556	-5.110946
H	4.121665	-0.727876	-5.188446
H	3.014025	-0.717314	-4.130088
O	1.351213	-2.173712	-6.412892
H	1.684776	-2.326888	-7.306389
H	2.044912	-1.617936	-5.955146
O	5.856305	-0.935639	-4.503860
H	6.027777	-0.157474	-3.891643
H	6.631348	-0.991744	-5.076716
O	1.162448	-4.353643	-4.603298
H	1.154335	-3.728350	-5.356836
H	0.299170	-4.826169	-4.564804
O	-1.271907	-5.563369	-4.060221
H	-1.512755	-6.419131	-4.436640
H	-2.113644	-5.031111	-4.044101
O	-1.212662	-0.853478	-6.297845
H	-1.007749	0.087851	-6.518445
H	-0.375277	-1.349408	-6.414997
O	-3.759333	-1.866348	-5.839665
H	-4.201177	-2.086220	-6.669565
H	-2.872144	-1.514517	-6.086171
O	-3.606827	-4.214763	-4.136879
H	-4.263871	-4.149679	-3.415631
H	-3.663690	-3.392321	-4.659573
O	4.590536	3.294856	-2.873498
H	3.794389	2.751167	-2.651933
H	4.319592	3.748714	-3.694519

O	2.958991	4.335013	-4.974046
H	3.243546	4.913153	-5.692785
H	2.311260	4.871895	-4.434627
O	6.384507	1.084115	-2.877919
H	6.513672	0.821459	-1.945363
H	5.834812	1.897384	-2.891525
O	2.054687	1.872866	-6.040424
H	2.389423	2.678556	-5.593058
H	2.501540	1.089180	-5.657927
O	-0.501871	1.773589	-6.628947
H	-0.565893	2.166066	-7.508886
H	0.471037	1.822801	-6.371939
O	1.295079	5.873424	-3.559660
H	1.563257	6.156300	-2.659785
H	0.323288	5.776665	-3.562811
O	-1.566562	5.726587	-3.800755
H	-2.142788	5.797151	-3.001453
H	-1.849114	6.431084	-4.397485
O	-2.285433	3.085162	-4.763179
H	-1.931147	3.952631	-4.494872
H	-1.652968	2.677746	-5.383514
O	6.208819	1.710464	2.013004
H	6.415123	1.138400	1.249046
H	5.896382	1.127909	2.734452
O	4.728607	3.962976	1.640945
H	4.397252	4.240162	2.522502
H	5.335810	3.192898	1.788560
O	7.000407	0.061737	-0.236986
H	7.965908	0.064629	-0.203360
H	6.731482	-0.889503	-0.179824

O	4.470924	5.342285	-0.832393
H	4.699496	4.948304	0.033910
H	4.585473	4.640120	-1.507575
O	2.235091	6.754185	-1.020344
H	2.561738	7.654309	-1.149027
H	3.061397	6.193875	-0.902989
O	3.572511	4.560884	4.167934
H	4.073512	5.195605	4.696738
H	2.675349	4.980695	4.042380
O	1.212037	5.813641	3.875403
H	0.943179	6.111957	2.984851
H	0.417920	5.425629	4.294884
O	0.306137	6.341836	1.121045
H	0.947992	6.650987	0.448975
H	0.286274	5.352462	0.993712
O	5.687353	-4.283847	1.926134
H	6.416227	-4.813646	2.273772
H	4.944260	-4.942087	1.742310
O	4.783217	-2.460376	3.992254
H	5.077398	-3.049517	3.268588
H	4.050777	-2.913174	4.452300
O	6.124980	-2.527283	-0.206421
H	6.140914	-3.246947	0.461791
H	6.051126	-2.913308	-1.119155
O	5.389177	0.167199	4.275326
H	6.167855	0.101596	4.843498
H	5.099605	-0.771415	4.131004
O	3.736010	2.066926	5.767669
H	3.627508	2.871125	5.225108
H	4.242890	1.433905	5.224063

O	2.661588	-3.905782	5.375360
H	2.216842	-4.456770	4.670789
H	3.064220	-4.529819	5.992553
O	0.674249	-2.053565	6.161790
H	1.437213	-2.664539	6.092102
H	1.011597	-1.169123	6.416623
O	1.305257	0.805627	6.350331
H	2.168607	1.262429	6.464919
H	1.258425	0.755597	5.361019
O	3.578431	-5.070010	-3.416464
H	3.941670	-5.838733	-3.874460
H	2.739745	-4.847085	-3.880623
O	2.592589	-6.252196	-0.910649
H	1.618820	-6.153465	-1.005370
H	2.978248	-5.784576	-1.671607
O	5.771538	-3.282007	-2.784774
H	5.755180	-2.498285	-3.369594
H	4.978712	-3.809847	-2.999770
O	3.785287	-6.056807	1.530312
H	3.361963	-6.131219	0.635903
H	3.056044	-6.047601	2.173966
O	1.555786	-5.262091	3.324283
H	0.613972	-5.519059	3.394331
H	1.583983	-4.568821	2.617025
O	-0.138760	-5.816712	-1.312867
H	-0.572388	-5.817749	-2.182297
H	-0.835498	-5.998988	-0.643797
O	-2.101444	-6.278952	0.620098
H	-1.826175	-6.117331	1.541459
H	-2.977922	-5.865443	0.512628

O	-1.318094	-5.820868	3.411438
H	-1.539525	-6.631699	3.887427
H	-1.955220	-5.132167	3.737011
O	-2.828791	0.275668	5.464346
H	-2.270076	0.947770	5.897637
H	-2.613215	-0.601643	5.859510
O	-2.206865	-2.233238	6.445969
H	-2.555251	-2.921579	5.849317
H	-1.238627	-2.343286	6.480327
O	-3.119358	-3.890645	4.199877
H	-2.907001	-3.088204	3.656561
H	-4.039128	-4.087587	3.934351
O	-5.142764	0.697783	4.240912
H	-4.260937	0.543052	4.688075
H	-5.772089	0.832955	4.961245
O	-3.314454	5.164570	3.406657
H	-3.040682	5.572914	2.567535
H	-3.984654	4.477915	3.181504
O	-1.133309	4.470657	4.860611
H	-2.011407	4.747642	4.488545
H	-1.261166	3.919382	5.654540
O	-1.030682	2.209868	6.742243
H	-1.209346	2.277652	7.688799
H	-0.145737	1.762637	6.667338
O	-5.194723	3.195793	2.805202
H	-5.127033	2.338433	3.269758
H	-5.515220	2.993919	1.905888
O	-5.542316	4.230264	-2.040070
H	-6.270890	4.756482	-2.393620
H	-5.298337	3.581328	-2.774528

O	-3.149436	5.640939	-1.570554
H	-3.036207	6.086360	-0.701361
H	-4.050752	5.249751	-1.637426
O	-2.388278	6.691278	0.930916
H	-1.393727	6.663229	0.971595
H	-2.642952	7.579230	1.212873
O	-5.927150	2.330477	0.103755
H	-4.997246	2.011433	-0.063017
H	-6.048940	3.027588	-0.570831
O	-6.926583	-1.749054	-2.160092
H	-6.992847	-1.204234	-1.349805
H	-6.469806	-1.190097	-2.824935
O	-5.181177	-0.261250	-3.800416
H	-4.521106	-0.453771	-3.084410
H	-4.830851	-0.717476	-4.588076
O	-4.899059	2.590406	-3.991048
H	-5.060648	1.625406	-3.938029
H	-3.979421	2.709395	-4.318709
O	-7.200476	-0.065577	0.182750
H	-6.797967	0.841658	0.099991
H	-8.142957	0.079999	0.337201
O	-5.658432	-3.909883	2.766246
H	-6.463749	-4.304691	3.123750
H	-5.851192	-2.928174	2.697506
O	-4.575314	-4.776020	0.313832
H	-5.141218	-4.665661	-0.480369
H	-5.090761	-4.539843	1.115672
O	-5.761186	-4.138314	-2.141886
H	-6.189619	-3.230972	-2.102314
H	-6.434046	-4.724353	-2.511994

O	-6.268951	-1.330617	2.614151
H	-5.810554	-0.654814	3.158751
H	-6.515707	-0.905319	1.769362
O	-0.038880	0.025202	0.183317
H	-0.152079	-0.936276	-0.035531
H	-0.090871	0.532465	-0.662155
O	-0.250129	-2.616525	-0.291727
H	0.331636	-3.059703	0.369731
H	-1.190002	-2.891281	-0.159922
O	-0.161363	1.451962	-2.213132
H	-0.785186	2.200323	-2.073417
H	-0.572457	0.817395	-2.844580
O	2.386763	0.256542	1.401544
H	1.514760	0.132213	0.948274
H	2.976705	-0.443969	1.031901
O	-2.134194	0.692589	1.845509
H	-1.822540	1.434305	2.417499
H	-1.341864	0.456163	1.303288
O	3.516476	-1.811005	0.105603
H	4.470287	-2.061565	0.077315
H	3.300438	-1.565618	-0.824622
O	0.617799	-2.411226	-2.722902
H	0.833815	-3.192851	-3.275833
H	0.300523	-2.709793	-1.825996
O	2.507009	1.790033	-2.032793
H	1.547015	1.843505	-2.251677
H	2.588213	2.148371	-1.110525
O	2.654142	2.706997	0.476308
H	2.678145	1.815350	0.912747
H	3.404749	3.226798	0.864774

O	-1.687551	3.339617	-1.090261
H	-1.009141	3.519171	-0.386896
H	-2.144702	4.182222	-1.296815
O	-2.877371	-2.692301	0.050843
H	-3.505512	-3.454668	0.073101
H	-3.170375	-2.049800	-0.639741
O	-3.457191	1.453594	-0.313338
H	-2.854318	2.174495	-0.630357
H	-3.074060	1.190118	0.567462
O	-1.214080	-0.665393	-3.520297
H	-0.553704	-1.369978	-3.283137
H	-1.304228	-0.697334	-4.495982
O	0.141202	-1.849973	3.462851
H	0.211056	-2.034309	4.427815
H	-0.801634	-1.939190	3.177285
O	1.586475	-3.205681	1.592000
H	2.360821	-2.812546	1.129562
H	1.244701	-2.565267	2.257238
O	2.813584	-0.884842	-2.432035
H	2.669321	0.068088	-2.204181
H	1.944703	-1.354691	-2.476918
O	1.065911	0.633371	3.696892
H	1.798683	0.590007	3.042865
H	0.612018	-0.239347	3.566283
O	-0.645413	2.575804	3.029703
H	-0.805742	3.249997	3.737264
H	0.027435	1.927432	3.357091
O	0.181040	3.714437	0.795501
H	1.099638	3.369427	0.644596
H	-0.124597	3.300196	1.651243

O	-2.421968	-1.874595	2.598260
H	-2.511594	-0.892878	2.583706
H	-2.614630	-2.155779	1.668594
O	-3.371097	-0.758709	-1.849860
H	-2.570139	-0.738360	-2.426703
H	-3.369130	0.079426	-1.316219
